# Treatment and Relapse Prevention of Typical and Atypical Optic Neuritis

**DOI:** 10.3390/ijms23179769

**Published:** 2022-08-29

**Authors:** George Saitakis, Bart K. Chwalisz

**Affiliations:** 1 Division of Neuro-Ophthalmology, Department of Ophthalmology, Massachusetts Eye & Ear Infirmary, Harvard Medical School, Boston, MA 02115, USA; 2 Athens Eye Hospital, 166 75 Athens, Greece; 3 Department of Neurology, Massachusetts General Hospital, Harvard Medical School, 15 Parkman Street, Suite 835, Boston, MA 02114, USA

**Keywords:** atypical optic neuritis treatment, typical optic neuritis, MOG, NMO, MS prevention

## Abstract

Optic neuritis (ON) is an inflammatory condition involving the optic nerve. Several important typical and atypical ON variants are now recognized. Typical ON has a more favorable prognosis; it can be idiopathic or represent an early manifestation of demyelinating diseases, mostly multiple sclerosis (MS). The atypical spectrum includes entities such as antibody-driven ON associated with neuromyelitis optica spectrum disorder (NMOSD) and myelin oligodendrocyte glycoprotein antibody disease (MOGAD), chronic/relapsing inflammatory optic neuropathy (CRION), and sarcoidosis-associated ON. Appropriate and timely diagnosis is essential to rapidly decide on the appropriate treatment, maximize visual recovery, and minimize recurrences. This review paper aims at presenting the currently available state-of-the-art treatment strategies for typical and atypical ON, both in the acute phase and in the long-term. Moreover, emerging therapeutic approaches and novel steps in the direction of achieving remyelination are discussed.

## 1. Introduction

Optic neuritis (ON) is an inflammatory condition involving the optic nerve but is far from being a uniform condition, and several important variants are now recognized that can be stratified into typical and atypical forms. Typical, ON usually manifests in young adults, especially women, between 18 and 45 years of age, and can be idiopathic or represent an early manifestation of demyelinating diseases, mostly multiple sclerosis (MS). The atypical spectrum includes entities such as neuromyelitis optica spectrum disorder (NMOSD), myelin oligodendrocyte glycoprotein antibody disease (MOGAD), chronic/relapsing inflammatory optic neuropathy (CRION), and sarcoidosis-associated ON, and in all of these, the clinical presentation, visual prognosis, and recurrence risk differ from typical ON. Importantly, optimal treatment approaches are also not uniform, making it essential to more accurately differentiate these entities based not only on their clinical presentation but also their pathogenesis. We will discuss currently available state-of-the art therapeutic strategies for typical and atypical ON, both in terms of acute treatment with regard to long-term relapse prevention. Moreover, emerging therapeutic approaches and novel steps in the direction of achieving remyelination are discussed.

## 2. Typical Optic Neuritis

Optic neuritis (ON) is an inflammatory condition involving the optic nerve, manifested usually in young adults, especially women, between 18 and 45 years of age [1]. The majority of the cases are idiopathic, but ON can be associated with demyelinating diseases, most commonly multiple sclerosis (MS). Optic neuritis represents one of the most frequent phenotypes of MS relapse, and occurs as the first demyelinating event in about one out of three MS patients [2]. MS is characterized by the presence of plaques that form in the CNS in combination with inflammation, demyelination, axonal injury, and axonal loss. The plaques are located primarily in the white matter of the brain, spinal cord, and optic pathways, but there is also involvement in the gray matter [3,4]. Depending on their stage of development, they contain varying proportions of immune cells and immunoreactive substances [5]. Plaques are expressed in all forms of MS, but vary over time quantitatively and qualitatively, showing a profound heterogeneity in the structure and immunopathological patterns of demyelination and oligodendrocyte pathology between relapsing-remitting and progressive forms of MS [6]. MS likely represents a T-cell-mediated autoimmune disorder with a predominance of CD8^+^ cells. The dominant theory is that inflammatory lesions in MS consist mainly of CD8^+^ and CD4^+^ T cells, and activated microglia and macrophages [7,8]. There is evidence regarding the suppression of functions that restricts CD4^+^ T-cell responses, and the tissue-damaging role of CD8^+^ T cells is reported to co-localize with axonal pathology [9,10]. Experiments in humanized transgenic mice showed that the specific interaction of CD8^+^ T cells with target cells requires MHC-I expression, which is tightly regulated in neurons and MHC-I molecules, only in response to danger signals such as pro-inflammatory cytokines IFN-γ or TNF-α [9]. However, the role of B cells has also become apparent, as evidenced, for instance, by the effectiveness of B cell inhibition as an MS disease-modifying therapy (DMT).

## 3. Pathophysiology of ON

In the acute phase, ON pathology is characterized by optic nerve abnormalities and inflammatory demyelination. More specifically, predominant T cell, B cell, and glial cell activation within the nerve increases pro-inflammatory cytokines, leading to the activation of microglia and monocyte-derived macrophages, and further recruitment of CD4^−^ and CD8^+^  T cells [11]. The subsequent inflammation leads to demyelination, reactive gliosis, and axonal death [12]. Pro-inflammatory cytokines and cytotoxic factors target myelin-producing oligodendrocytes (OLGs) and oligodendrocyte precursor cells (OPCs), causing apoptosis and exacerbating axonal demyelination [13,14,15,16]. Mature OLGs that survive demyelination are unable to produce new myelin sheaths. Remyelination, therefore, requires the migration and regeneration of oligodendrocytes from OPCs [17].

It is worth noting that the acute inflammatory lesions of the afferent visual pathway cause retrograde degeneration of retinal ganglion cells (RGCs). It has been demonstrated that RGC loss is associated with a reduction in post-synaptic proteins and neurite projections, and with persistent microglia and astroglia activation in the inner retina with high levels of iNOS (inducible nitric oxide synthase), IL (interleukin)-1α, TNF (tumor necrosis factor)-α, and C1q (complement component 1q) [15]. Thus, the development of therapeutic agents should focus on anti-inflammatory, anti-apoptotic, and remyelinating mechanisms to achieve neuroprotection and neuro-regeneration in the optic nerve and retina.

## 4. Acute Treatment of Typical Optic Neuritis/Clinically Isolated Syndrome

In general, MS is characterized by its tendency for recurrence in proximity to a previously affected site, as has been observed radiologically [18,19] and confirmed in post-mortem pathological studies [20]. Lotan et al. showed that in MS, recurrent episodes of ON tend to attack the same optic nerve that was affected before [21]. Similar findings come from a 2011 study [22]. Potential explanations for the recurrent nature of ON in MS is the disruption of the blood–brain barrier during the initial insult and antigenic change and expansion, leading to epitope spreading as a pathogenic event leading to a chronic CNS demyelinating disease [23].

Based on the presence of prominent immunologic activity in the pathologic samples of MS patients and oligoclonal bands in the CSF of most MS patients, it has been suggested that the disease is an immune-mediated disorder [6,24,25,26]. However, there are alternative theories claiming that MS is not a homogenous condition, thus not fulfilling the criteria of an autoimmune disease [27,28,29]. Much effort has been invested in identifying the autoantigen(s) against which the oligoclonal bands are directed, so far without success. It is believed that the inflammatory attack is not an outcome of an immune response directed against a specific auto-antigen. Thus, in MS, unlike NMOSD and MOG antibody disease, the immune response may be nonspecific and triggered by tissue changes induced by the previous attack.

Corticosteroid use has traditionally been the common approach for the treatment of ON, with the first implementation dating back to the 1950s [30]. Data from the United States demonstrate that the majority of ophthalmologists and neurologists in the 1980s used to treat their patients with optic neuritis with standard oral doses of corticosteroids, despite the lack of convincing evidence of efficacy [30,31]. The Optic Neuritis Treatment Trial (ONTT) was the first multicenter, randomized, collaborative clinical trial of ON [30,31]. Fifteen centers in the United States participated in the ONTT, recruiting 457 patients between 1 July 1988 and 30 June 1991. Patients were enrolled who had acute unilateral optic neuritis with visual symptoms lasting 8 days or less, aged between 18 and 45 years, with no previous history of optic neuritis in the affected eye, no evidence of associated systemic disease other than MS, and no previous treatment with corticosteroids for MS or optic neuritis [31]. The mean age of patients at study entry was 32 years, 77% of patients were women, and 85% identified as white. The participants were randomized either to be treated with oral prednisone (1 mg/kg daily for 14 days), intravenous methylprednisolone (250 mg every 6 h for 3 days) followed by oral prednisone (1 mg/kg daily for 11 days), or oral placebo. Each regimen was followed by a short oral dosage taper consisting of 20 mg of prednisone (or placebo) on day 15 and 10 mg of prednisone (or placebo) on days 16 and 18 [29,30]. In general, steroid treatment was well tolerated, with only minor adverse effects (sleep disturbance, mild mood change, upset stomach, facial flushing, mild weight gain), except for a case of acute transient depression and another patient that suffered from acute pancreatitis. Patients were evaluated in seven follow-up visits during the first 6 months, at 1 year, then yearly through 1997, in 2001 through 2002, and finally in 2006. According to the study design, the primary outcome for the treatment group comparison was set at 6 months. 

The study findings demonstrated that the natural course of visual functions after an episode of typical optic neuritis, either treated or untreated, is one of a rapid visual recovery beginning within 2 weeks after the onset of symptoms, with most of the recovery often taking place after 4 to 6 weeks, and further slow recovery over several months, even up to 1 year [2]. In almost all patients, regardless of the treatment group and initial severity of visual losses, some improvement began within the first 30 days [2,30]. Of clinical relevance, recurrences of optic neuritis occurred more commonly in patients treated with oral prednisolone alone; within 2 years from diagnosis, the probability of recurrence in either eye was almost 2-fold higher in the low-dose prednisone group (30%) than in either the placebo group (14%) or the high-dose intravenous group (16%) [30,31,32,33]. The ONTT showed that vision recovered faster in the intravenous group than in the other groups, although the difference among the three groups had faded by 30 days. However, at 6 months, qualitative features such as contrast sensitivity, visual field, and color vision were still slightly better in the intravenous group. By contrast, the prednisone group compared with the placebo group demonstrated no significant differences in the rate of recovery or the 6-month outcome for any aspect of the visual function. At the 6-month point, patients in all three treatment groups had a median visual acuity of 20/16, and fewer than 1 out of 10 patients had a visual outcome of 20/50 or worse. At the 1-year follow-up, there was no statistically significant difference in visual function among the groups. Visual acuity was 20/40 or better in 95% of the placebo group, 94% of the intravenous steroid group, and 91% of the oral steroid group at 1 year. After 15 years, 72% of the eyes affected with optic neuritis had visual acuity of ≥20/20, and 66% of the patients had ≥20/20 acuity in both eyes [1]. A 2015 Cochrane Systematic Review also reported the failure of intravenous steroids to improve vision outcomes in ON [34]. The ONTT also found that among the 389 patients without a diagnosis of clinically probable or definite MS at study entry, the intravenous steroid group showed a lower rate of development of clinically definite MS within the first 2 years (7.5%) than did the placebo (16.7%) or prednisone (14.7%) groups, but this apparent protective effect was not sustained at 3 years [30]. By 5 years, the treatment had no significant effect on the development of MS. Most of the aforementioned intravenous treatment group benefits on the development of MS were observed in patients with brain findings on the magnetic resonance imaging (MRI) at baseline, because the rate of MS among patients without baseline MRI lesions was so low that therapeutic efficacy could not be determined. 

Some potential limitations of the trial include the definition of symptom onset (timed from the visual loss but not from the onset of pain), inclusion of possible MOG cases, the validity of the primary outcome measure of high-contrast visual acuity, and the lack of pharmacokinetic data (making it difficult to develop a plausible biological explanation for as to why oral vs. intravenous corticosteroids should be harmful compared with intravenous corticosteroids). In addition, the long interval between the onset of symptoms and initiation of treatment in ONTT (up to 8 days) leaves open the possibility that a “critical time window” may have been missed, and that more vision loss could be prevented if treatment was initiated in the early inflammatory phase (within 48 h) [35,36]. Experimental evidence supports such a critical time window for treatment initiation in optic neuritis, as it has been shown that inflammation of the optic nerve precedes demyelination and axonal degeneration by about 2 days, and irreversible damage to the axonal cytoskeleton occurs within 5–7 days [35,37]. Indeed, a retrospective study demonstrates significant improvement in both functional and structural outcomes in patients with relapsing ON when treatment is initiated early [38]. 

The current standard of care for typical optic neuritis, still based on the results of the ONTT, is either no treatment in mild cases or the administration of intravenous steroids to accelerate visual recovery [30,39,40]. A proton pump inhibitor may also be given to prevent peptic ulcers. There is no role for low-dose oral prednisone [31]. This reasoning is consistent with a Cochrane meta-analysis as well [41]. 

Since the publication of the ONTT, other studies have shown that high-dose oral corticosteroids and high-dose IV methylprednisolone are bioequivalent, and have similar effects on MRI outcomes and clinical MS relapse [2]. Morrow et al., in 2018, showed in a single-blind randomized clinical trial that the efficacy of high-dose oral steroids is bioequivalent to and shows no inferiority to intravenous steroids. More specifically, 55 participants were randomized to either methylprednisolone sodium succinate (1000 mg, IV) daily for 3 days or oral prednisone (1250 mg) daily for 3 days. Improvements in vision were noticed at 1 month and at 6 months [2]. Compliance with this oral regimen has been previously shown to be very high [2]. Similar results were cited by the COPOUSEP trial in France [42]. In addition, a Cochrane review in 2008 compared the efficacy of the two forms of steroid administration and found them to be equally effective. Studies have also shown that intravenous dexamethasone in a dose of 200 mg/day had comparable efficacy to 1 g/day of intravenous methylprednisolone, and has the advantage of low costs and fewer side effects [39]. Intramuscular or subcutaneous adrenocorticotropic hormones are also approved for the treatment of ON- and MS-related relapses [40]. 

Intravenous immunoglobulin (IVIg) has a potential role in the management of acute optic neuritis, though evidence is limited, and the agent is typically reserved for the treatment of patients with steroid-refractory ON. IVIg may cause rash, fever, and, rarely, aseptic meningitis, thrombosis, hemolysis, and renal dysfunction [39]. In general, plasma exchange (PLEX) is typically favored over IVIg to manage MS relapses that are not responsive to steroid treatments. PLEX is associated with a number of potential side effects including myocardial infarction, arrhythmia, hemolysis, central line placement risk, and death in a small percentage of patients [43]. More recently, high-dose cyclophosphamide (50 mg/kg per day for 4 consecutive days, followed by a granulocyte-colony-stimulating factor 6 days after completion) was evaluated in nine patients with aggressive RRMS as a rescue treatment for acute fulminant relapses. Potential side effects of the short-term high-dose cyclophosphamide monotherapy in patients with MS include neutropenia and infection [40,44].

## 5. Long-Term Treatment: Immune Prophylaxis against Optic Neuritis Relapses/Progression to Multiple Sclerosis

### 5.1. Mechanisms of Action in Interferon β in MS and Optic Neuritis

**Figure 1:**
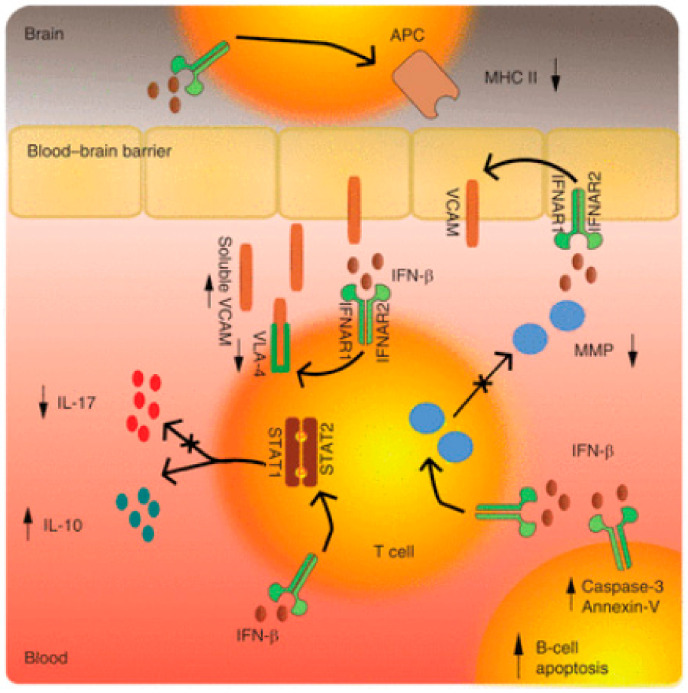

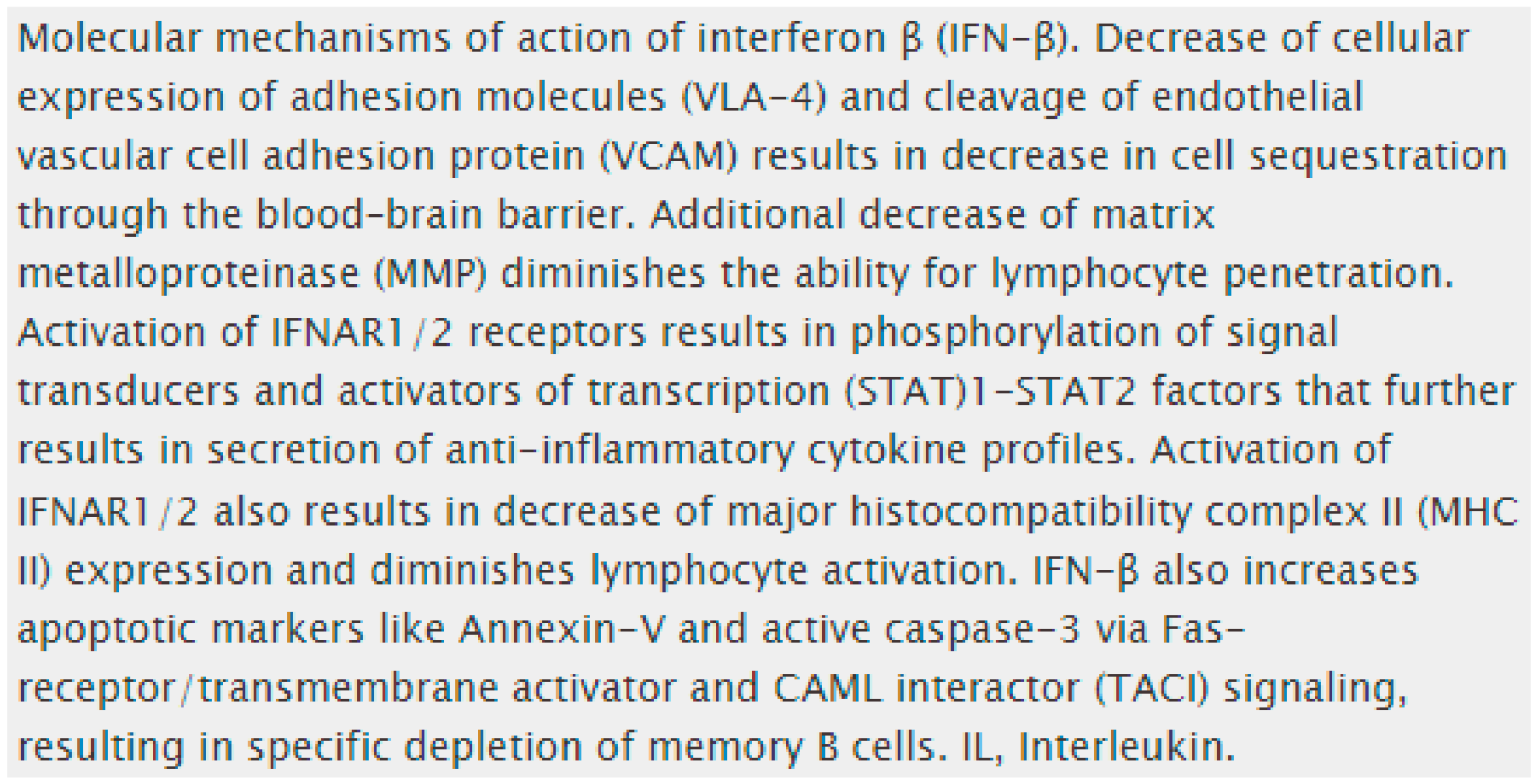
Molecular Mechanisms of Action of Interferon β [45].

Interferons (IFNs) have been recruited as a potential therapeutic option for MS based on their immunomodulatory and antiproliferative properties [45]. It is believed that IFNs act via several overlapping mechanisms such as the down-regulation of the major histocompatibility complex (MHC) class II expression present on the antigen-presenting cells, the induction of T-cell production of interleukin 10 (IL-10), and thus a shift in the balance toward anti-inflammatory T helper (Th)-2 cells, and the inhibition of T-cell migration as a result of a blockade of metalloproteases and adhesion molecules [46] (Figure 1: a synopsis of IFN mechanisms of action). 

The actions of IFNs are mediated through transcriptional factors and subsequent gene regulation. The major route in which IFN-β produces its effect is by activating the Janus kinase (JAK) signal transducers and activators of the transcription (STAT) pathway. More specifically, IFN-β binding to the type I IFN receptor causes phosphorylation of STAT1 and STAT2 and the formation of STAT1-STAT2 heterodimers, which translocate to the nucleus, bind the IFN-stimulated response element (ISRE), and modulate the expression of ISRE-regulated genes [47]. It has been demonstrated that the cellular response to IFNs is complex and results in changes in the expression of more than 500 genes representing ∼0.5% of the human genome [48]. Rizzo et al., focusing on the pivotal role of B cells in MS immunopathology, investigated the mechanism of B-cell apoptosis. The up-regulation of mechanisms that require FAS-receptor/TACI (transmembrane activator and CAML interactor) signaling and the production of apoptotic markers such as Annexin-V and caspase-3 were shown as specific inducers of B-cell apoptosis [49]. 

### 5.2. Glatiramer Acetate (GA)

**Figure 2:**
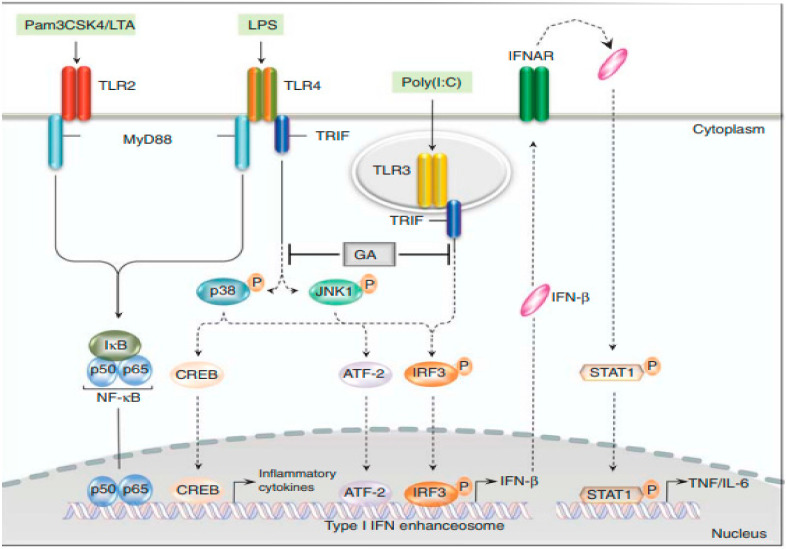

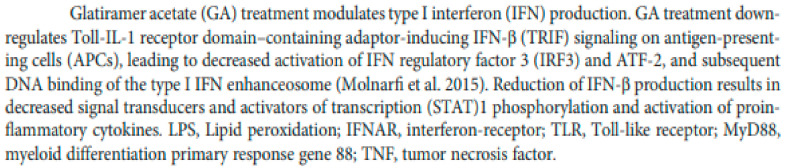
Glatiramer Acetate Modulates Type I Interferon production [50].

**Figure 3:**
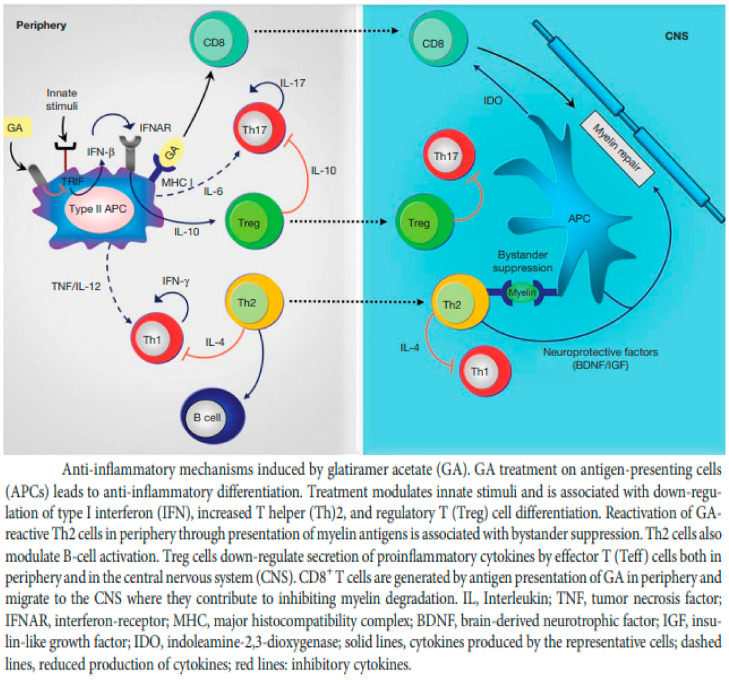
Anti-inflammatory Mechanisms Induced by Glatiramer Acetate [52].

The mechanism of action of GA has long been an enigma. GA has well-established immunomodulatory properties, promoting the expansion of anti-inflammatory and regulatory Th2 and Treg cells and inducing the release of neurotrophic factors. Using various genetically modified mouse strains, as well as human monocytes, Molnarfi et al. showed that GA inhibited the TRIF-dependent pathway, resulting in a reduction in IFN-β production [50] (Figure 2). This observation is consistent with the earlier demonstration that STAT1 phosphorylation is reduced upon activation in type II monocytes [51]. These findings provide a key anti-inflammatory mechanism connecting innate and adaptive immune modulation in GA therapy. Animal studies have also shown that GA-reactive Th2 cells migrate to the CNS and accumulate at the site of active lesions. Thus, GA-reactive T cells provide the effector arm in treatment. However, GA treatment influences both innate and adaptive immune compartments, and it is now recognized that antigen-presenting cells (APCs) are the initial cellular targets for GA, and it is the modulation of the APC compartment to anti-inflammatory (M2) phenotypes that leads to an expansion in regulatory Th2 and Treg cells. In addition, the anti-inflammatory (M2) APCs induced following treatment with GA are responsible for the induction of anti-inflammatory T cells that contribute to its therapeutic benefit [52]. Mechanisms of action of GA that promote immunomodulation and neuroprotection are not mutually exclusive, and several may contribute to the efficacy of the drug (Figure 3). 

### 5.3. Treatment of Clinically Isolated Syndromes

In this article, we are reviewing some of the trials that specifically addressed clinically isolated syndromes such as optic neuritis. These are mostly older trials. We will not cover all multiple sclerosis treatments in detail, but acknowledge that several newer DMTs for MS have class I evidence for MS, and have approval for treatment of both MS and CIS. This evidence (and the FDA approval of these medications) is based on MS trials, not specifically CIS/optic neuritis trials, and will not be reviewed in detail. Thus, in clinical practice, a number of additional MS medicines may be used for high-risk CIS patients, likely with good efficacy, although they were not specifically investigated in the CIS situation. It is beyond the scope of this review to discuss all such treatment options. 

The goal of MS treatment is to delay the onset of additional clinical relapses and possibly long-term disability. The first opportunity to initiate disease-modifying therapy in patients with MS may actually be when they are in the clinically isolated syndrome (CIS) stage, i.e., before conversion to clinically definite MS (CDMS). Since 1993, when interferon beta-1b was approved for MS, a growing number of disease-modifying therapies (DMTs) have become available. The goal of DMTs is to decrease the frequency of clinical relapses, lessen the number of new and active multiple sclerosis lesions on MRI, and, in the long term, to slow the progression of neurologic impairment. Since the approval of natalizumab as the first highly active DMT, the ultimate goal of “no evidence of disease activity” (NEDA) has become attainable for many patients. While the treatment of MS is beyond the scope of this review, the evidence for initiating MS DMTs after CIS is discussed.

Most DMTs approved for MS are also approved for the treatment of CIS. However, only a few DMTs have specifically been evaluated in clinical trials to treat CIS (including ON) and to delay the onset of clinically definite MS, including interferons and glatiramer acetate [53,54,55,56,57,58]. In all trials, the patients who received the active drug developed a second neurologic manifestation (definite multiple sclerosis) less frequently, and (if at all) at a later time, than those given the placebo. Even after a second episode, treated patients had a significantly lower annual rate of relapse for the duration of the follow-up period. Neurologic impairment was generally relatively mild and not significantly different between the two groups. 

Interferons and glatiramer acetate have been approved for the treatment of CIS, including ON with two or more inactive typical lesions of multiple sclerosis on MRI. CHAMPS (Controlled High-Risk Subjects Avonex Multiple Sclerosis Prevention Study) was a randomized, double-blind trial involving 383 patients with an initial, acute monosymptomatic demyelinating event—unilateral ON, incomplete transverse myelitis (TM), or brainstem/cerebellar—and at least 2 silent T2 lesions on brain MRI [59]. The patients were randomized to weekly intramuscular interferon β-1a (IFN-b1a) or a placebo. The treatment group experienced a 44% reduction in the rate of development of CDMS compared with the placebo group over 3 years of follow-ups. There were statistically significant beneficial effects on all MRI parameters for the treatment group, including a decrease in T2 lesion development, gadolinium-enhancing lesions, and T2 lesion volume. The 10-year follow-up showed that patients treated immediately after their first episode had a significantly lesser chance of experiencing a second attack compared to those who had delayed treatment. Based on these results, FDA extended its approval of intramuscular IFN-b1a to include patients with CIS deemed to be at high risk for MS. The most common side effects associated with interferons are flu-like symptoms, including myalgia, fever, fatigue, headache, chills, nausea, vomiting, pain, and asthenia [59].

The PRISM (Prevention of Relapses and Disability by Interferon β-1a Subcutaneously in Multiple Sclerosis) trial assessed the efficacy of interferon (IFN)-β1a compared to the placebo, in dosages of 22 μg and 44 μg given subcutaneously in relapsing-remitting MS patients; both treatment groups had fewer relapses [60]. The Early Treatment of Multiple Sclerosis (ETOMS) trial showed that weekly subcutaneous IFN-β1a reduced the conversion to CDMS over 2 years to 34% vs. 45% for the placebo; a post hoc analysis found that the treatment group had a reduced rate of brain atrophy compared with those on the placebo [61]. The BENEFIT (Betaseron in Newly Emerging Multiple Sclerosis for Initial Treatment) study included patients with a single neurologic event and at least 2 clinically silent MRI lesions; in a 24-month study period, the standard dose of IFN-β1 was seen to reduce the risk of MS by 50%. Furthermore, open-label extension studies from the original CHAMPS and BENEFIT cohorts have suggested a possible long-term benefit from the early initiation of disease modifying treatments [62]. The CHAMPIONS (Controlled High-Risk Avonex Multiple Sclerosis Prevention Study in Ongoing Neurologic Surveillance) trial concluded that a delay in treatment by up to 3 years after a first clinical demyelinating attack could lead to an earlier time for CDMS but did not show a long-term effect on the development of new MRI T2-weighted lesions or long-term disability [63]. The REFLEX (REbif FLEXible dosing in early MS) trial evaluated 517 patients with CIS and at least two clinically silent T2 lesions on brain MRI. At two years, the probability of MS diagnosed by the McDonald criteria was significantly lower with subcutaneous interferon β-1a 44 mcg dosed either three times a week or once a week (63 and 76 percent, vs. 86 percent for the placebo). In the subsequent extension phase of the trial, all patients (*n* = 403) received interferon β-1a. At five years, the group assigned to interferon β-1a treatment in the placebo-controlled phase (i.e., early treatment) continued to have a reduced probability of conversion to MS and fewer new MRI lesions compared with the group whose treatment was delayed for up to two years [64,65,66].

Glatiramer acetate is an immunomodulator used to reduce relapse frequency in relapsing–remitting multiple sclerosis [39]. The PreCISe (Early GA Treatment in Delaying Conversion to CDMS in Participants Presenting with a Clinically Isolated Syndrome) trial showed a reduced conversion to CDMS (25%) in patients treated with 20 mg of glatiramer acetate subcutaneously daily compared to 43% for the placebo [63]. 

Teriflunomide also reduces the risk of progression to multiple sclerosis, as has been shown in the TOPIC (Teriflunomide Vs. Placebo in Patients With First Clinical Symptom of Multiple Sclerosis) trial, where 618 adults with a CIS were randomly assigned in a 1:1:1 ratio for treatment with 14 mg of oral teriflunomide daily, 7 mg of teriflunomide daily, or the placebo for up to 108 weeks, with a median treatment duration of over 70 weeks. The agent reduced the risk of relapse-defining CDMS at both the 14 mg dose and the 7 mg dose. The exact mechanisms by which teriflunomide works in MS are not established; it is an oral dihydroorotate dehydrogenase inhibitor that interferes with de novo synthesis of pyrimidines and thus inhibits the proliferation of rapidly dividing cells such as autoreactive T and B cells [64]. The most common adverse effects of teriflunomide were elevated alanine aminotransferase (ALT) levels, diarrhea, hair thinning, paresthesia, and upper respiratory tract infections. Teriflunomide is associated with increased risk for hepatotoxicity and teratogenicity and should not be given to patients with liver disease or women who are pregnant. Full immunization coverage is required prior to treatment initiation [53,54,55,67]. In addition, intravenous immune globulin and minocycline have been studied for the treatment of CIS or the first demyelinating event, but are not established as effective [56,57,58].

The early treatment of CIS is not favored by all experts. The decision whether to initiate treatment for CIS has to consider that not all patients go on to develop any additional relapses or lesions, and that the evidence base showing that the early treatment of CIS will prevent long-term disability is very limited. Patients should be informed of the potential benefits, risks, and uncertainties, and participate in decision making [62]. However, once a diagnosis of CDMS is made, the early initiation of treatment is recommended. 

## 6. Emerging Therapeutic Approaches

### 6.1. Remyelination/Recovery from Optic Neuritis

After an acute episode of optic neuritis, GCL complex loss may start as early as 8 days after onset, and RNFL thinning has been reported as early as after 1 month, predicting optic atrophy at month 6. Recovery from relapses in MS patients involves remyelination of white matter and optic nerve lesions after the recruitment and differentiation of oligodendrocyte precursors from the lesion perimeter, but it is limited by axonal degeneration and glial scarring, which are observed even at the earliest stages of the disease. The currently available DMTs have neither neuroprotective effects nor the potential to enhance remyelination; thus, a crucial therapeutic gap exists. Recently, the effectiveness of the sphingosine-1 phosphate receptor (S1PR) modulator fingolimod in promoting remyelination after a first unilateral episode of acute optic neuritis was evaluated in a phase 2 study [68]. Since S1PR are required for lymphocytes to exit lymphatic follicular structures, fingolimod exerts immune modulation by sequestering pathogenic T- and B-cells from the blood stream. Importantly, S1PR are also present on neurons, astrocytes, and oligodendrocytes, as well as resident and CNS-invading myeloid cells, where they were shown to mediate neuroprotective and pro-regenerative effects in preclinical studies [68]. Fingolimod readily crosses the blood–brain barrier. Fingolimod was associated with a better recovery from unilateral optic neuritis compared to treatment with IFN-β 1b, and could have a role as an early treatment by promoting remyelination, preventing astrogliosis, and preserving axons [68].

Recent prospective studies have evaluated novel therapeutic approaches for neuroprotection and remyelination in acute optic neuritis. In 2017, opicinumab, a human monoclonal antibody against leucine-rich repeat and immunoglobulin domain-containing neurite outgrowth inhibitor receptor-interacting protein-1 (anti-LINGO-1), was investigated in the RENEW trial as a potential remyelinative therapy in acute ON [69,70]. It was hypothesized that the agent would enhance remyelination by directly promoting the proliferation and differentiation of oligodendrocyte precursors. LINGO-1 blockade has no detectable immunomodulatory effects. Treatment with opicinumab produced no significant change in the visual evoked potential (VEP) latency at 24 weeks (a measure of remyelination) in the intention-to-treat population; however, significant improvements in VEP latency delay were observed at 24 and 32 weeks in the prespecified per-protocol patient population. Since anti-LINGO-1 treatment had no differential effect on anatomic measures of optic nerve fiber loss, i.e., retinal nerve fiber layer (RNFL) or ganglion cell complex (GCC) thickness in either the intention-to-treat or per-protocol patient population at 24 weeks (with a mean delay of 24 days between ON onset and the start of treatment), the authors suggested that therapeutic windows may be longer for remyelination compared to axonal neuroprotection. The antiepileptic and proposed neuroprotectant phenytoin, studied in a Phase 2 randomized controlled trial, was shown to decrease RNFL loss in acute ON; however, no effect on visual outcomes or VEPs was found [69,70].

As a promising emerging therapy, mesenchymal stem cell (MSC) therapy has been suggested to be capable of stimulating both the remyelination and neuroprotection of axons in other neuro-degenerative diseases and in animal models of ON. In addition, cell-free approaches utilizing extracellular vesicles (EVs) produced by MSCs are considered to be a viable alternative to the transplantation of stem cells. EVs secreted by living cells mainly include exosomes and microvesicles. MSCs are amongst the largest cellular producers of EVs. Recent studies have shown that EVs can accommodate intracellular communication and act as modulators of cellular immunity, cancer biology, and regeneration/remyelination. Importantly, EVs can pass through the blood–brain barrier (BBB), making them suitable for CNS treatment. EVs exhibit anti-inflammatory and neuroprotective effects in multiple animal models of neuro-degenerative diseases and in rodent models of MS [1,71,72,73]. In particular, MSC-derived EVs are involved in a wide variety of physiological processes including the inhibition of natural killer cells, B cells, and mitogen-activated T cells, moderating microglia and macrophage polarization and reducing oxidative stress. In addition, they show the potential of tissue regeneration and myelin membrane biogenesis [74]. Studies in experimental autoimmune encephalomyelitis (EAE) mice have yielded evidence that EVs attenuate neuroinflammation and demyelination by reducing and downregulating T-cells (Tregs, CD4+), macrophages, astrocytes, and microglia. The immunomodulatory effect may be mediated by promoting a shift in microglial phenotypes from M1 (pro-inflammatory) to M2 (anti-inflammatory) [75]. MSC-EVs may also promote axon remyelination by protecting oligodendrocytes and their precursor cells from damage caused by immune cells [76].

**Figure 4:**
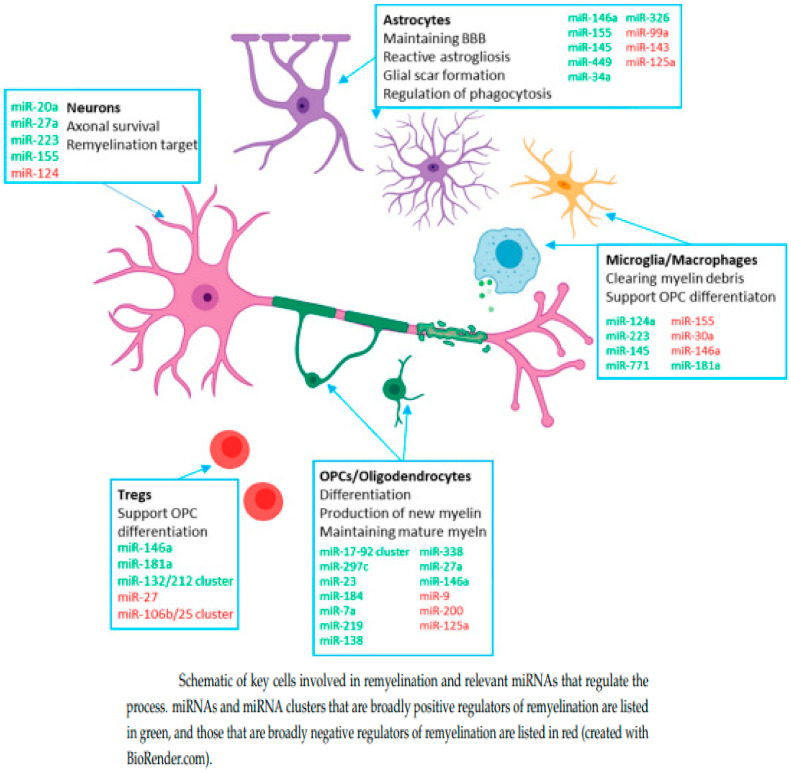
Key Cells involved in Remyelination and relevant miRNAs [77].

MicroRNAs appear to mediate most EV effects. The pathology of MS is influenced by histone modifications and gene regulation via microRNAs [77,78]. MicroRNAs mediate post-transcriptional gene silencing and are involved in cellular activities including proliferation, differentiation, and migration, as well as disease initiation and disease progression [77,78], Figure 4. A series of studies in MS patients and animal models demonstrate that various types of microRNA (microRNA-219, microRNA-125a-3p, mir-27a) may be involved in the regulation of oligodendrocytes [79]. Exosomes or viral vectors can play a role as carriers of miRNAs to therapeutically regulate MS pathology. In addition, the overexpression of proteins that modulate exosomal miRNA gene expression profiles have the potential to improve the therapeutic effects of exosomes [77,78]. 

### 6.2. Atypical Optic Neuritis

Atypical Optic Neuritis includes entities in the demyelination diseases’ spectrum such as ON associated with neuromyelitis optica spectrum disorder (NMOSD), which is associated with aquaporin 4 antibodies (AQP4), and myelin oligodendrocyte glycoprotein antibody disease (MOGAD), which is associated with myelin oligodendrocyte glycoprotein (MOG) antibodies. These diseases differ from typical and MS-related ON in clinical features, visual morbidity, and therapeutic approaches.

### 6.3. NMOSD

In 2004, the discovery of a pathogenic NMO-associated IgG antibody, targeting the water channel membrane protein aquaporin-4 (AQP4), was an important milestone in differentiating NMO from MS [80]. AQP4 is highly concentrated on astrocyte end feet in the CNS. All NMO lesions show a widespread and early loss of AQP4 immunoreactivity, in contrast to MS lesions, where AQP4 immunoreactivity is often increased [81,82]. The binding of AQP4-ab to astrocyte AQP4 channels triggers classical complement cascade activation, followed by granulocyte, eosinophil, and lymphocyte infiltration, culminating in injury first to astrocytes then to oligodendrocytes, and demyelination, neuronal loss, and neurodegeneration [83]. In humans, AQP4 monomers are expressed in astrocytes in two isoforms: M1-AQP4 and M23-AQP4. Both isoforms have identical extracellular domain residues, but M1-AQP4 has 22 more amino acids at the cytoplasmic N terminus. However, AQP4-ab binding to the ectodomain of astrocytic AQP4 has isoform-specific outcomes. M1-AQP4 is completely internalized, whereas M23-AQP4 resists internalization and is aggregated into larger-order orthogonal arrays of particles (OAPs), a process facilitated by M1-AQP4 deficiency [84]. Alterations in OAPs are required for NMO-IgG to recognize conformational AQP4 epitopes, as well as for the binding of the complement component C1q to clustered AQP4-ab [85,86]. CNS lesions in NMOSD patients are characterized by IgG, IgM, and complement deposits with a rosette pattern, most prominent around vessels, as well as cellular infiltrates of granulocytes (neutrophils and eosinophils) macrophages/microglia and T cells. The key feature is AQP4 loss on astrocytes. In certain lesions however, other typical astrocytic markers, such as glial fibrillary acidic protein (GFAP) and S-100β, are still detectable, indicating AQP4 loss precedes astrocyte death. Ultimately, the preservation or secondary loss of neurons and the associated demyelination will depend on disease severity. Demyelination affects both gray and white matter, sometimes with necrosis, cavitation, and thickened, hyalinized vessels. Thus, the autoimmune response in NMOSD primarily affects astrocytes and is initiated by the autoantibody-mediated loss of AQP4 [87].

The visual outcome after NMOSD-ON is less favorable compared to MS-ON and MOGAD, supported by an increased thinning of RNFL and GCL complex in NMOSD cases [44,88]. Therefore, early and aggressive treatment is appropriate in the acute phase of NMOSD-ON. The ONTT did not enroll any NMOSD patients, and its findings are not applicable to NMOSD-ON. Given the devastating nature of NMOSD-ON, no treatment is not an option, and steroids alone may be insufficient in many cases [88]. Given the recurrent nature and devastating morbidity of relapses, disease-modifying therapy should be instituted early after the diagnosis of NMOSD is established [88,89].

### 6.4. Acute NMOSD Relapses

Relapses are usually treated with 1 g of high-dose **IV methylprednisolone (IVMP)** daily for 3–7 days, followed by oral steroid tapering. The likelihood of complete visual recovery increases when IVMP is administered within 5 days of the onset of NMOSD ON [36]. However, Kleiter et al. demonstrated, in their analysis of NMOSD optic neuritis, an incomplete efficacy of IVMP, as only 33% of patients achieved complete remission [90]. Furthermore, repeated courses of IV steroids only reduced the number of non-responders. Patients with optic neuritis and concurrent myelitis had an even worse prognosis [90].

**IVIg** and **PLEX** are immunomodulatory therapies that may offer additional benefits for acute optic neuritis treatment in NMOSD. Clinical trials have suggested a range of potential improvements in visual functions from 45–55%; however, due to their retrospective design, they failed to define criteria for the optimal use or timing of PLEX [46]. There was also mostly no distinct interval between the completion of IVMP and the institution of PLEX, rendering debatable whether clinical improvement results from PLEX induction or from delayed IVMP effects. Features such as male sex, lower baseline disability, rapid initiation of treatments, and shorter relapse durations have been associated with a greater response to PLEX. According to one study, 50% of patients with poor visual recovery after high-dose intravenous steroids (<20/200 or less) recovered a visual acuity of at least 20/30, with a mean time to PLEX initiation of 30 days [91]. The reasoning for this treatment in NMOSD is based on the fact that most of the astrocyte and neural destruction is caused by the deposition of AQP4-IgG and the subsequent complement activation. PLEX removes circulating antibodies, complements, and cytokines from the blood, which may shorten the action of antibodies and lessen further inflammation and necrosis [92]. Some retrospective studies of NMOSD have shown that the very early concurrent initiation of PLEX or immunoadsorption with corticosteroids during acute relapses may improve outcomes [90,93,94,95]. However, even delayed PLEX therapy may still be a reasonable treatment option for patients with acute refractory ON [89]. PLEX may be accompanied by serious side effects such as hypotension, infection, hypocalcemia, and coagulopathy [93]. In addition, several authors have described the use of monthly or yearly PLEX sessions to avoid relapses in NMOSD patients. It seems that the removal of the humoral autoimmunity, in addition to the modulation of cellular inflammation by IVMP, may increase the interval between relapses [96,97,98].

**Immunoadsorption** represents an alternative form of therapeutic apheresis, currently not approved in the United States. It uses modified membranes to achieve the selective removal of antibodies from plasma, allowing for the removal of the pathogenic autoantibodies while sparing other plasma proteins, therefore eliminating the need for protein replacement and potentially minimizing complications. Immunoadsorption has been reported to benefit steroid refractory ON and NMOSD-ON [93]. 

As an additional therapeutic solution, in a retrospective study of 10 NMOSD patients unresponsive to IVMP, IVIg was effective in 50% of patients [87]. Recently, a retrospective study demonstrated the superiority of high-dose IVMP plus IVIgG treatment compared to a high dose of IVMP alone [99]. 

When the aforementioned interventions fail to salvage visual functions, immunosuppression with intravenous cyclophosphamide may represent an avenue of final resort. A subset of NMSOD patients with acute TM seem to have benefited from this treatment [100]. Outside of case reports, no clinical studies have been published on the response of severe ON to intravenous cyclophosphamide, and the treatment is not without risk. 

### 6.5. NMOSD Relapse Prevention

In contrast to MS, in NMOSD, functional decline and the development of disability are related primarily to relapses [101]. After acute stabilization, the early institution of long-term preventative and maintenance immunosuppressive therapies is needed to minimize permanent visual and neurologic disability [101]. So far, no standard management has been agreed upon for first-line treatment or treatment switching [87]. Since June 2019, there are now three new monoclonal antibodies FDA-approved for treating AQP4-Ab-seropositive NMOSD patients, targeting three different disease pathways, based on efficacy in phase III randomized controlled trials. Prior to this, the most commonly used conventional maintenance/disease modifying therapies were rituximab, azathioprine, and mycophenolate mofetil (MMF) used off label. Immunosuppressive therapies such as methotrexate, mitoxantrone, and cyclophosphamide have been shown to be beneficial in highly active NMOSD, but are infrequently used due to their less favorable risk–benefit profiles [102]. Low-dose corticosteroids have not been systematically studied but are frequently used, either as maintenance therapy or as an add-on to conventional immunosuppressants [103]. There is also a level 2 recommendation for hematopoietic stem cell transplantation (HSCT) in refractory courses (106, 107, CAMPUS; NCT04064944).

**Azathioprine** and **MMF** are agents with broad immunosuppressive properties which have been used, based on retrospective studies or uncontrolled case series published before 2019, as effective first-line treatments for NMOSD, either as a monotherapy or in conjunction with low-dose corticosteroids [103]. The agents have demonstrated efficacy, with a significant reduction in the annual relapse rate and the stabilization or improvement of EDSS scores [87]. For full biologic effects to be observed, AZA and MMF require at least 4–6 months of treatment, rendering oral steroid co-administration advisable to provide an immunosuppressive bridge from treatment onset [101]. In contrast to rituximab, the immunomodulatory effects of AZA and MMF are mediated by the rather unselective suppression of fast-dividing immune cells [104]. Retrospective comparisons among these agents are subject to confounding by indication and other biases and have produced mixed results. 

MMF is a noncompetitive inhibitor of inosine monophosphate dehydrogenase, an enzyme essential for de novo synthesis of the purine nucleotide guanosine-5′-monophosphate, which inhibits the proliferation of lymphocytes [105,106,107]. MMF is a semi-synthetic derivative of mycophenolic acid (MPA), which is the active metabolite of MMF. MPA acts as a selective noncompetitive inhibitor of inosine 5-monophosphate dehydrogenase type II, which is a rate-limiting enzyme in the de novo synthesis of guanine ribo- and 2-deoxyribonucleotides. MPA has a mean terminal half-life of 17 h and has been shown to prevent the production of interferon gamma (INF-γ), lipopolysaccharide-induced interleukin-6 (IL-6), and oxidative stress [108]. At a cellular level, MPA depletes the guanosine pool in lymphocytes and inhibits T- and B-cell proliferation/transendothelial migration, macrophage activation, dendritic cell functioning, and immunoglobulin production [109]. 

AZA is a prodrug form of 6-mercaptopurine (6-MP), which was first introduced in clinical practice in the 1960s for kidney transplantation to prevent immunological rejection. The agent is converted non-enzymatically to 6-MP, which is metabolized in the liver to the active metabolite 6-thioinosinic acid and works as a purine antagonist that gives negative feedback on purine metabolism and inhibits DNA and RNA synthesis. Its action results in the inhibition of T-cell activation, a reduction in antibody production, and a decrease in the levels of circulating monocytes and granulocytes [110]. 

Among the benefits of AZA treatment are the convenient oral administration and the affordability of the agent compared to rituximab [106]. Recently, data from 150 NMOSD patients treated with AZA showed that 69% had no accumulation of disability after a 5-year follow-up [111]. A retrospective study evaluating 103 AQP4-IgG-seropositive NMOSD patients demonstrated that 89% of patients had a significant reduction in median ARR from 1.5 to 0, 61%, remained relapse-free at a median follow-up of 18 months, and neurological functions improved or stabilized in 78% of patients with azathioprine treatment [106]. Unfortunately, treatment was discontinued in the last follow-up for 46% of patients due to side effects in 62% (increased liver enzymes and pancytopenia). Many patients discontinue AZA over time, raising the concern of poor tolerability [106]. Common side effects include bone marrow suppression with consequent pancytopenia and hepatitis, and viral infections. Intolerance is not uncommon as well. More rarely, pancreatitis and severe gastrointestinal disturbances can occur [106]. An increased risk of malignancies has been shown, with lymphoma development in 3% of patients in a large NMOSD series [107]. Patients on AZA should be monitored regularly with complete blood count, liver, and renal function tests [106]. 

**Figure 5:**
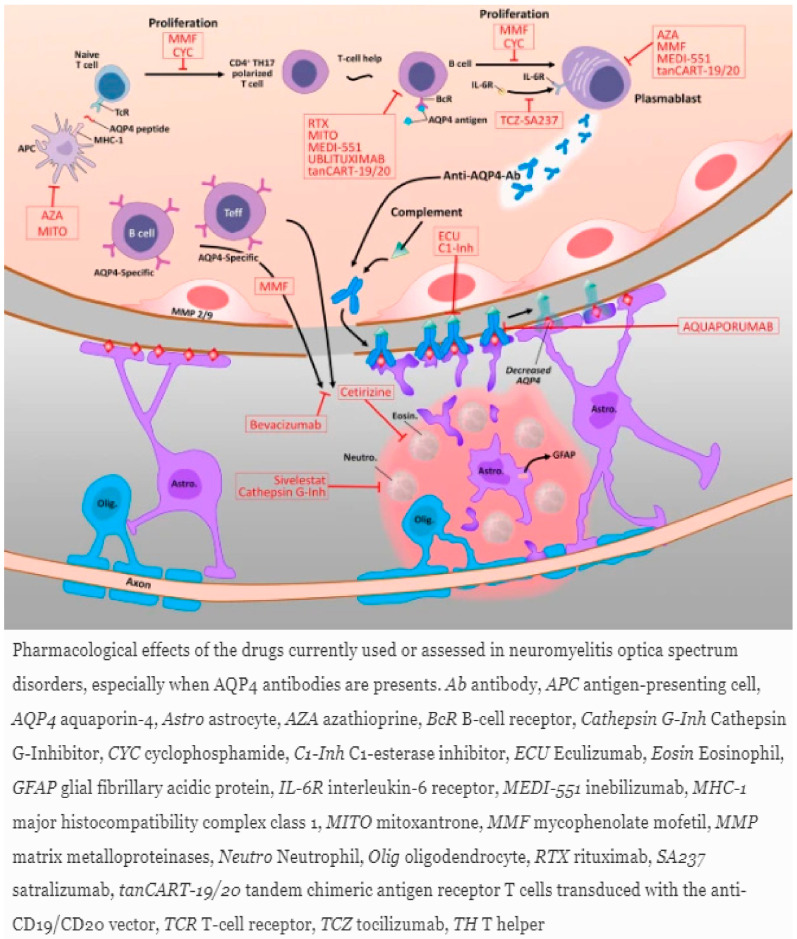
Pharmacological Effects of the drugs used in NMOSD [115].

**Figure 6:**
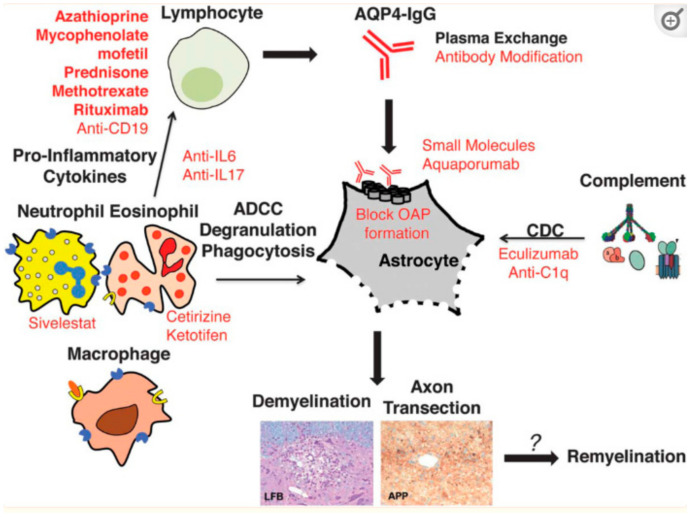

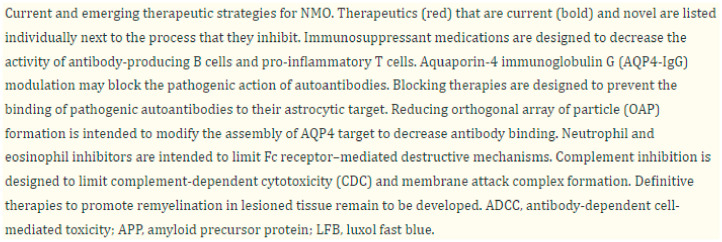
Current and Emerging Therapeutic Strategies for NMO [116].

MMF seems effective in doses of 1750 mg to 2000 mg per day and may be used in conjunction with prednisone [107]. In 2009, Jacob et al. showed in a case series of 24 patients with NMOSD the effectiveness of the agent. The median dose of MMF was set at 2000 mg per day for a median duration of 27 months. A total of 79% had an improvement in ARR, and disability was stabilized or improved in 91%. One died of disease complication during follow-up, and 25% had to discontinue MMF treatment due to side effects including headache, constipation, easy bruising, anxiety, hair loss, diarrhea, abdominal pain, and leukopenia [112]. More recently, a study reported 50.7% of patients experience a relapse on MMF, 59.7% continued on MMF, and 83% showed a stabilization or improvement in their disability at the most recent follow-up [113]. In addition, among 28 patients treated at the Mayo Clinic and the Johns Hopkins Hospital with MMF, failure rate was 36%, similar to that of rituximab and better than for azathioprine [114]. Case series and a meta-analysis suggest that the efficacy of mycophenolate mofetil is comparable to rituximab, and mycophenolate mofetil was more tolerable in meta-analyses [107,114]. Known adverse effects of MMF include an increased risk of lymphoma in transplanted patients and nonmelanoma skin carcinomas, infections (viral and bacterial), gastrointestinal symptoms (ulcers, hemorrhages), and cytopenia [106,114]. Teratogenicity represents a major concern with the need for contraception in young female patients in their reproductive age, as congenital malformations have been reported in 26% of live births, and the risk of first-trimester pregnancy loss is 45% in exposed patients [114]. Figure 5 and Figure 6 demonstrate the mechanisms of action of agents utilized in the treatment of NMOSD.

**Methotrexate** is a folate derivative which inhibits dihydrofolate reductase and nucleotide synthesis. Traditionally, it has been used in weekly oral doses in the treatment of autoimmune diseases such as rheumatoid arthritis and Crohn’s disease. The evidence for methotrexate in NMOSD comes from small observational studies, the largest of which included 14 AQP4-IgG-seropositive patients followed for a median of 21.5 months, demonstrating an improvement in ARR ranging from 64% to 100%, and a relapse freedom in 22% to 75% of patients [108,114]. Patients should be monitored for bone marrow suppression and liver functioning. The most common side effects are bone marrow suppression and impaired liver functions, while rare, serious complications include pneumonitis, aplastic anemia, and opportunistic infections. Methotrexate is a teratogen. 

Alternative broad-spectrum immunosuppressive agents include mitoxantrone, cyclophosphamide, cyclosporine A, and tacrolimus. A systematic review in 2019 identified 8 studies with 117 NMOSD patients treated with the agents [117]. The majority of the studies reported a significant improvement 6 months to 5 years following treatment in terms of ARR [117]. **Mitoxantrone** is a topoisomerase II inhibitor impairing DNA repair, resulting in a drop in B and T cells. A comparison study of NMOSD treatments demonstrated the inferiority of mitoxantrone to rituximab and azathioprine/prednisolone with regard to the relapse rate [118]. Mitoxantrone has been associated with severe adverse events, such as dose-limiting cardiotoxicity and an increased risk of acute myeloid leukemia, especially in patients having received a cumulative dose greater than 60 mg/m^2^ [107]. **Cyclophosphamide** is an alkylating agent that crosslinks guanine bases in DNA. There is controversy with regard to its effectivity in NMOSD patients. Data from Brazil showed relapses in six out of seven patients treated with pulse doses of cyclophosphamide [119]. In contrast, a recent retrospective study of 41 patients treated for a median of 13.6 months reported a median ARR drop from 0.7 to 0.0 [120]. Mitoxantrone and cyclophosphamide are teratogenic. A report in 2013 of nine seropositive NMOSD patients treated with **Cyclosporine A** showed a decrease in ARR from 2.7 to 0.4 [113]. Cyclosporine A is a calcineurin inhibitor that binds to cyclophilins, resulting in the inhibition of the translocation of transcription factors, leading to a reduced transcriptional activation of several cytokines and ultimately to reduced T cell proliferation [106]. Potential side effects include hypertension, nephrotoxicity, tremor, opportunistic infections, and increased hair growth. **Tacrolimus** is also a calcineurin inhibitor, which reduces peptidyl-prolyl isomerase activity by binding to immunophilin FKBP-12 and leads to the inhibition of T lymphocyte signal transduction and IL-2 transcription. It is an orally administered agent, widely used in organ transplantation and systemic autoimmune diseases. A Chinese retrospective study of 25 patients with NMOSD treated with 2 to 3 mg/d of tacrolimus, and concomitant prednisone in 60% of patients, found that tacrolimus decreased the ARR by 86% and improved the EDSS from 4.5 pretreatment to 2.3 at the last follow-up [121]. In addition, another study in Japan of patients with NMOSD showed that the initiation of prednisolone followed by tapering doses of prednisolone and tacrolimus in 25 patients, dosed with 1 to 6 mg/d, achieved relapse freedom in 92%, with relapses only seen in patients with subtherapeutic serum concentrations [107]. Serious side effects associated with the use of the agent include severe infections and malignancies. Hyperglycemia, diabetes mellitus, hyperkalemia, nephrotoxicity, and tremors have been also described [107,121].

A potential role for **long-term intermittent IVIg** in preventing relapses in NMOSD has been suggested, as there is evidence that IVIg is effective in reducing the relapse rate and improving neurological disability in NMOSD patients. One case series treated 8 NMOSD patients (2 seropositive) with IVIg (0.7 g/kg/day for 3 days, 4–21 infusions per patient) for a mean duration of 19 months, demonstrating a remarkable decrease in the mean ARR and the EDSS score as well. In addition, a study where IVIg (0.4 g/kg/day for 5 days, then 0.4–1.0 g/kg/day every 2 to 3 months) was given to six NMOSD patients (4 seropositive) for an extended mean duration of 4 years confirmed the favorable results in terms of median ARR improvement, while 50% of the patients were relapse-free at a 4-year-follow-up. In conclusion, IVIg could be considered a safe alternative in NMOSD patients with repeated infections from immunosuppressant therapy; however, controlled trials are required to confirm efficacy [106].

**Rituximab** has been used to prevent relapse in NMOSD on an off-label basis for more than 15 years [102]. The agent is recommended as a first-line maintenance treatment of NMOSD in the 2010 guidelines from the European Federation of Neurological Societies and the 2014 recommendations of the Neuromyelitis Optica Study Group [122]. It is a chimeric monoclonal antibody that rapidly leads to marked CD20+ B cell depletion via complement-mediated and cell-mediated cytotoxicity. In addition, there is evidence that rituximab in AQP4-IgG-seropositive patients leads to a predominance of B regulatory cells after therapy [58]. B cell depletion lasts, on average, 6–9 months [106]. A meta-analysis in 2016 on 25 studies re-demonstrated the efficacy and safety of Rituximab in NMOSD patients regarding the annual relapse rate (ARR) and qualitative indices [102]. Importantly, a prospective study of 100 NMOSD with a long follow up of 7 years showed that 94% of patients experienced a significant reduction in ARR, and 70% were relapse-free while on rituximab [123]. In comparative studies, rituximab has shown its superiority to AZA and MMF in decreasing annual relapse rates and relapse severity as well as preventing new relapses [87,103]. A common therapeutic approach is the administration of an induction dose of 1000 mg of rituximab once or repeated twice 2 weeks apart, followed by a fixed a fixed regimen of 1000 mg of rituximab every 6 months [124]. Alternative approaches include a dosing regimen based on body mass index, administering 375 mg/m^2^ per week for 4 weeks, or an individualized dosing scheme on the basis of CD19+ lymphocytes reemergence [102]. 

It is worth noting that in Japan, rituximab for NMOSD has been covered by insurance from June 2022. Recently, Tahara et al. conducted the RIN-1 study in Japan, the first multicenter, randomized double-blind placebo-controlled Phase III time-to-event clinical trial of rituximab in NMOSD [125]. AQP4-antibody-positive patients with an EDSS of 7.0 or less were randomized 1:1 to receive either rituximab intravenously (375 mg/m^2^ of body surface for week 1 to 4, then 1000 mg i.v. at week 24, 26, 48, and 50) or with a matching placebo and concomitant oral prednisolone, which was tapered over the study’s duration of 72 weeks. No other immunosuppressants were allowed. None of the patients treated with rituximab relapsed, in contrast to 37% on the placebo [125].

In long-term rituximab therapy, however, 15–45% of patients continue to have relapses. This may potentially be related to the early repopulation of B cells, or to the sequestration of tissue-resident B cells outside the blood stream [126]. Alternative theories include the presence of neutralizing antibodies against rituximab, polymorphisms in the FCGR3A-F allele, and CNS compartmentalization of pathogenic B cells that may also interfere with effective B cell depletion by the agent [102].

Rituximab use can result in the development of hypogammaglobulinemia in a significant portion of patients (20–65%), especially with prolonged therapy, and an increased risk for severe infections, including herpes zoster, tuberculosis, and recurrent sino-pulmonary and urinary tract infections [127]. Hepatitis B, active tuberculosis, and other severe infections need to be excluded or treated before the initiation of treatment. In cases of severe hypogammaglobulinemia, an inadequate response to vaccines, and/or frequent or severe infections, the supplementation of IVIG 400 mg/kg every 4 weeks targeting a serum level 9 of 800–1000 is recommended [127,128]. Infusion reactions are common and can usually be managed by pretreatment with intravenous steroids, antihistamine, and slow infusion [107]. In addition, there have been rare cases of progressive multifocal leukoencephalopathy (PML) following rituximab therapy in rheumatoid arthritis, but none has been reported in NMOSD [129]. 

**Tocilizumab** is a humanized monoclonal antibody against the IL-6 receptor, which has been approved for the treatment of rheumatoid arthritis, giant cell arteritis, juvenile idiopathic arthritis, and cytokine release syndrome; however, it is not FDA licensed for NMOSD [130]. The rationale for tocilizumab use in NMOSD is based on the involvement of IL-6 in the pathophysiology of disease [130,131]. IL-6 promotes an increased bloodbrain barrier permeability with the infiltration of proinflammatory cytokines and antibodies into the CNS and the survival of a plasmablast population responsible for secreting anti-AQP4 antibodies, leading to increased AQP4-IgG production in vitro and ex vivo [130,131]. IL-6 represents the only cytokine that is found in higher levels in the serum and the cerebrospinal fluid of patients with NMOSD compared with MS controls [107,130]. 

A series of case reports has documented the effectiveness of tocilizumab in NMOSD, including patients refractory to rituximab, since 2013 [107]. For example, three patients with aggressive AQP4-IgG-seropositive NMOSD uncontrolled by other immunosuppressants and completely CD19-depleted by rituximab, when switched to tocilizumab, showed an ARR decrease from 3.0 to 0.6, though without improvement in clinical disability [106]. In addition, a pilot study with seven NMOSD patients who had experienced multiple relapses in the preceding year on immunosuppressants and corticosteroids, and were treated consequently with intravenous tocilizumab, reported a fall in mean ARR from 2.9 to 0.4, with five of seven participants achieving relapse freedom for at least 1 year. In another observational study of eight patients treated with tocilizumab as an add-on therapy for NMOSD, it showed remarkable effectivity in reducing the relapses by 90% compared with the baseline [107,130]. In 2019, a study of 12 NMOSD patients treated with subcutaneous tocilizumab also demonstrated the effectiveness of agent 37. Potential side effects of tocilizumab include a modest increase in lipoproteins, bowel perforation, and a higher risk of neutropenia and infections, such as tuberculosis, invasive fungal infections, and bacterial infections, the latter mainly with concomitant methotrexate. However, opportunistic infections are less likely to occur in NMOSD compared to rheumatoid arthritis [107,130]. 

In 2020, the TANGO trial was the first head-to-head prospective, randomized comparison study between an established and new therapeutic agent in NMOSD. It was a phase 2, open label, time-to-event study in China that compared the safety and efficacy of tocilizumab and azathioprine in NMOSD patients [132]. The tocilizumab group included 59 patients (85% AQP4 seropositive), and the azathioprine group 59 patients (90% AQP4 seropositive). Tocilizumab was administered at 8 mg/kg IV every 4 weeks, with concomitant immunosuppressive coverage for the first 12 weeks of treatment. Azathioprine was given initially, at an oral dose of 25 mg daily and increased by 25 mg per day to a target of 2–3 mg/kg/day, with immunosuppressives for the first 6 months of treatment. Analysis of the primary outcome of the time to first relapse favored tocilizumab over azathioprine, with a median of 78.9 weeks for tocilizumab vs. 56.7 weeks for azathioprine. Relapse occurred in 14% of the tocilizumab group and 47% of the azathioprine group. In the subgroup analysis of patients with concomitant autoimmune diseases, 9% in the tocilizumab group and 35% in the azathioprine group relapsed. In contrast, no differences were noticed in the risk of relapse among patients without concomitant autoimmune diseases. Regarding disability progression at 3 months, tocilizumab demonstrated a more favorable profile compared to azathioprine (8% vs. 25%) [132]. Furthermore, AQP4-IgG levels dropped by 50% in the tocilizumab group and remained unchanged in the azathioprine group. In seronegative patients, relapse occurred in 22% with tocilizumab and 50% of patients on azathioprine. Overall, adverse events were equally frequent, but some serious adverse events including the elevation of alanine transferase and upper respiratory and urinary tract infections were more common in the azathioprine group compared to tocilizumab. There was one death in each group, but neither death was treatment-related [132]. The authors concluded that tocilizumab significantly reduced the risk of relapse compared with azathioprine in NMOSD, proposing the agent as a potentially effective and safe treatment for relapse prevention in NMOSD [107,132].

**Figure 7:**
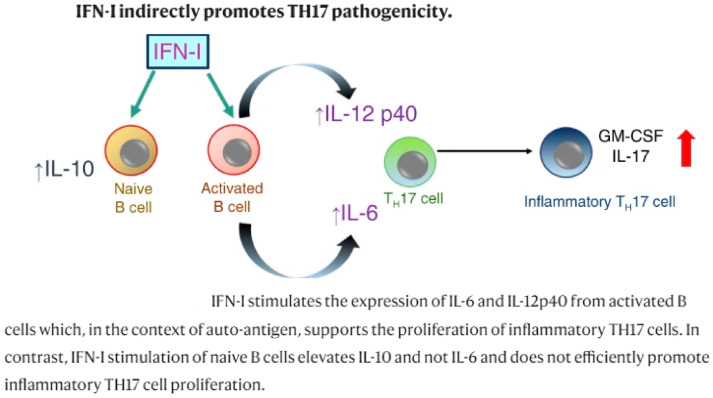
INF-I and TH17 pathogenicity [133].

**MS Therapies.** It is worth noting that some agents used in MS such as interferon, natalizumab, and fingolimod have been shown to not benefit or have a detrimental impact in AQP4-antibody-positive NMOSD. More specifically, IFN-β increases the relapse rate and promotes severe exacerbations, possibly by increasing the production of BAFF and IL-17 [133]—Figure 7. In addition, natalizumab, an antibody against very late antigen 4, has been reported to have no effect or to worsen disease activity in NMOSD patients either seropositive or seronegative. The proposed mechanisms of exacerbation involve florid active demyelination, severe neutrophilic and eosinophilic infiltrates, and severe astrocyte loss. The increase in the numbers of peripheral proinflammatory T cells or eosinophils can lead to eosinophil migration to the CNS, resulting in a surge in lesion formation or the stabilization of AQP4-specific bone marrow plasma cells. Furthermore, oral fingolimod has the potential to accelerate NMO disease activity; fulminant disease may develop early on after the initiation of therapy. A theory similar to natalizumab has been suggested, with fingolimod promoting bone marrow egress of eosinophils, triggering enhanced lesion activity and AQP4-IgG production.

### 6.6. FDA-Approved Disease-Modifying Therapies for NMOSD

**Eculizumab** is a humanized monoclonal IgG2/IgG4-hybrid antibody targeting C5, which inhibits cleavage and thus prevents the release of pro-inflammatory C5a and the involvement of C5b (the terminal complement component) in the membrane attack complex (MAC). Consequently, eculizumab could have dual action downregulating adaptive and innate immune responses either through C5a in the periphery (decreasing the chemotaxis of leukocytes to the inflammatory sites) or through C5b on astrocytes in the CNS [134]. Pathological analyses in NMOSD patients with acute lesions have shown both the early and specific involvement of the CNS vasculature and the crucial role of the complement in pathogenesis, demonstrating extensive, perivascular complement activation [130]. Eculizumab has been approved by the FDA as a treatment to prevent relapse in AQP4-IgG-seropositive adults with NMOSD since 2019, followed by the European Union and Japan. Of note, all patients who are to start eculizumab must receive the meningococcal vaccination at least 2 weeks before the first dose, since blocking the complement system increases the risk of infection with encapsulated bacteria. However, meningococcal vaccines do not fully protect against meningococcal disease, and concomitant antibiotic therapy can be considered [87,135]. Additional limitations on the widespread use of the agent include the frequent dosage scheme of bimonthly intravenous infusions and the high cost [87]. 

The efficacy of eculizumab in the prevention of relapse in NMOSD was initially suggested in 2013 by an open-label phase II trial of 14 AQP4-IgG-seropositive NMOSD patients with a highly active disease (55 attacks in 2 previous years in total). A total of 12 patients were relapse-free, and none progressed, 2 patients had possible attacks during twelve months on eculizumab, whereas 5 relapsed within five months after withdrawal. One patient who had received prior immunization suffered meningococcal sepsis and sterile meningitis during the treatment, and another one a fatal myocardial infarction (deemed unrelated) during follow-up. The PREVENT trial in 2019 was a phase 3, randomized, double-blind, placebo-controlled, time-to-event study of 143 AQP4-seropositive patients with NMOSD with EDSS less than or equal to 7 and a highly active disease (at least two relapses in the prior year or three in the prior 24 months) who were randomized 2:1 to eculizumab 900 mg, IV weekly × 4 doses followed by 1200 mg every 2 weeks or a placebo [136]. Patients were allowed to continue their prior immunosuppressive therapies, which occurred in 76% of cases. Patients who had been recently treated with rituximab, mitoxantrone, IVIg, and prednisone >20 mg per day, or were suffering from active bacterial infections, were excluded from the trial. Forty-six patients had previously used rituximab, which was stopped within three months before inclusion. In addition, all of the participants were vaccinated against Neisseria meningitidis before receiving treatment. The primary endpoint was the first adjudicated relapse. Given the uncertainty of when the final relapse would occur, the sponsor terminated the trial after 23 of the predefined 24 adjudicated relapses. Clinical relapse occurred in 3% of patients in the eculizumab group and 43% of patients in the non-eculizumab group, resulting in a 94% relative-risk reduction. In a subset analysis of patients who were on concomitant immunosuppression, 4% of the eculizumab group and 54% of the non-eculizumab group of patients experienced a relapse. However, there was no significant difference in disability progression. One patient on eculizumab and azathioprine died from pulmonary empyema, with cultures yielding Peptostreptococcus micros and Streptococcus intermedius [137]. During the open-label extension trial involving 137 patients, serious adverse events were reported in 36% of treated patients, including two cases of sepsis and one case of Neisseria gonorrheae infection, but no deaths. Furthermore, there was a higher rate of upper respiratory tract infections and headache in the eculizumab arm, but there were no cases of meningococcal infection [107].

**Inebilizumab** was the first B-cell-depleting agent to be approved by the FDA for the treatment of AQP4-IgG-seropositive NMOSD patients in June 2020. Prior to this, B cell depletion with the anti-CD20 agent rituximab had been used for off-label NMOSD treatment. However, rituximab does not deplete plasmablasts, which do not express CD20 [102,124]. Inebilizumab is an afucosylated humanized IgG1κ, anti-CD19 monoclonal antibody that directly binds CD19 with high affinity on the surface of B cells, which demonstrates dual action on B cell depletion through antibody-dependent cellular cytotoxicity and antibody-dependent cellular phagocytosis. Cytotoxicity specifically is enhanced via the process of afucosylation, which leads to a dramatic increase in the affinity of inebilizumab for FcγRIIIA, a receptor that mediates antibody-dependent cytotoxicity. CD19 expression on B cells begins at the pro-B stage. The wider expression of CD19 compared to CD20 on cells that constitute the B-cell lineage allows inebilizumab to target a broader range of pathogenic B cells not being targeted by anti-CD20 agents. Additionally, CD19-positive plasmablasts circulating in the peripheral blood of individuals with NMOSD may produce AQP4-IgG antibodies [138]. 

The N-Momentum trial (2019) was a phase 2/3, randomized, double-blind, placebo-controlled, time-to-event study of AQP4-seropositive and AQP4-seronegative patients with NMOSD. A total of 230 adults with active NMOSD were enrolled, defined as at least one attack requiring treatment the year before enrollment or two attacks in 2 years and an EDSS of 8 or less [139]. A total of 91% of the participants were women with a mean age of 43, and 92% of the patients were seropositive for AQP4 antibody. Exclusion criteria included treatment with rituximab or other B-cell-depleting agents within the previous 6 months, previously receiving a bone marrow transplant or T cell vaccination therapy, IVIg within the previous 1 month, natalizumab, cyclosporin, methotrexate, mitoxantrone, cyclophosphamide, tocilizumab, or eculizumab within the previous 3 months, or previous alemtuzumab or total lymphoid irradiation. About 70% of participants had had prior exposure to disease-modifying therapies. Patients were randomized 3:1 into the inebilizumab group (74–92% seropositive) or placebo group (56–93% seropositive). Interestingly, of the 17 AQP4-seronegative patients, 7 had antibodies against MOG. The patients were treated with 300 mg of inebilizumab IV or a placebo on days 1 and 15. Furthermore, all participants were given 20 mg of prednisone daily or an equivalent dose of other glucocorticoids between days 1 and 14, and then tapered through day 21 to minimize the risk of relapse at treatment initiation. Patients were not concomitantly treated with other immunosuppressive therapies. In the active group, a maintenance dose of 300 mg of inebilizumab was administered every 26 weeks. The double-blinded period lasted up to 197 days, until a new NMOSD attack, or until the termination of enrollment. All patients were thereafter offered open-label therapy. Because of a clear demonstration of efficacy, enrollment was stopped before reaching the target of 252 patients and 67 adjudicated attacks. Relapse occurred in 12% in the inebilizumab arm and in 39% in the placebo group (73% relative risk reduction). In the subgroup analysis of patients who were AQP4 seropositive, relapse occurred in 11% in the inebilizumab group and in 42% in the placebo group (hazard ratio 0.23). Due to the sample’s inequality regarding seronegative patients among groups (only four participants were randomized to the placebo arm), efficacy could not be interpreted in the seronegative subset. Of note, the trial also confirmed that the efficacy of inebulizumab was consistent across the clinical presentations of myelitis and optic neuritis domains [138,139,140].

The secondary endpoints remarkably showed that patients treated with inebilizumab had a significantly reduced likelihood to experience optic neuritis compared to the placebo arm (10 patients in each group); however, there were no differences in changes in the low-contrast visual acuity binocular score from the baseline among the groups. Additionally, the treated arm demonstrated a considerable reduction in the numbers of B cells (less than 10% of baseline) and the maintenance of low counts during the trial. The immunological effects of inebulizumab were observed within 4 weeks after the initiation of treatment. Furthermore, among AQP4-seropositive patients, fewer had a statistically significant worsening of their EDSS score. The inebilizumab arm also had lower numbers of cumulative active MRI lesions and NMOSD-related hospitalizations compared with the placebo [139,140,141]. Serious adverse events were similar among both the inebulizumab (9%) and placebo groups (5%); however, 2% of patients on the agent developed transient grade 3 neutropenia. There were no malignancies observed during the study. No death occurred during the placebo-controlled phase, but two patients died during the open-label phase. The first one was initially randomized on the placebo and passed away by respiratory insufficiency after a severe NMOSD attack preceded by pneumonia, and his death was considered unrelated to the treatment. The second patient, originally receiving inebilizumab, developed new neurological symptoms (weakness, aphasia, neurological decline, seizures) 9 days after receiving the maintenance dose. MRI showed new large lesions in white and grey matter, considered not representative for progressive multifocal leukoencephalopathy (PML), although one of three PCR tests on CSF was positive for JC virus, and brain biopsy was not performed; ultimately, the possibility that the death was treatment-related could not be excluded [140,141]. 

Inebilizumab is contraindicated for patients with active hepatitis B and active or untreated latent tuberculosis. Inebilizumab can also cause hypogammaglobulinemia, resulting in recurrent or serious opportunistic infections, which may require the discontinuation of the treatment or IVIg administration. Additionally, B-cell-depleting therapies in general are associated with an increased risk for malignancy and infection, including PML [107,138]. 

**Satralizumab** is a humanized IgG2 monoclonal antibody which binds membrane-bound or soluble interleukin 6-receptors, preventing the IL-6-induced inflammatory cascade. The pharmacokinetics of the agent have been optimized compared to its predecessor via an enhanced “antibody-recycling” process allowing for a longer half-life than tocilizumab. Satralizumab is designed to dissociate, pH-dependently, from the satralizumab-IL6-R complex within the endosome and to be recycled for repeated antigen binding in the peripheral blood, extending the interval of re-administration. Satralizumab is the third and most recent agent (2020) approved by the FDA for the treatment of adult patients with AQP4-IgG-seropositive NMOSD, including by self-injection [103,142,143]. In Japan, the agents are licensed for the treatment of both adults and children. Satralizumab is administered subcutaneously at weeks 0, 2, and 4, and then monthly, with instructions on withholding treatment in the event of an active infection, elevated liver enzymes, or neutropenia, and is contraindicated in patients with hepatitis B and active or untreated latent tuberculosis [107,144]. 

The safety and efficacy of satralizumab were evaluated in the SAkuraSky and SAkuraStar trials, phase III, randomized, double-blind, placebo-controlled, time-to-event studies of AQP4-seropositive (70%) and AQP4-seronegative patients (30%) with NMOSD [142,143]. In the SAkuraSky trial, patients on prior immunosuppressive therapies continued these treatments at stable doses (rituximab was excluded). In the SAkuraStar trial, the investigators compared only satralizumab monotherapy to the placebo without the use of concomitant immunosuppressive therapies. The therapeutical approach was either 120 mg of satralizumab subcutaneously or the placebo at weeks 0, 2, and 4, and then every 4 weeks. Inclusion criteria for the SAkuraSky trial included adolescents (age of at least 12 years) and adults, diagnosis of NMOSD by the 2006 criteria, history of at least two relapses in the previous 2 years with at least one relapse in the previous 12 months, and an EDSS score of 6.5 or less. In contrast, the SAkuraStar trial only included adults with the same prerequisites needed to be met. For both trials participants were excluded if they had received treatment with rituximab within the previous 6 months, eculizumab or multiple sclerosis disease-modifying therapies within the previous 6 months, anti-CD4 agents, cladribine, or mitoxantrone within 2 years, or IL-6 targets, alemtuzumab, total-body irradiation, or bone marrow transplantation in the past [142,143].

In the SakuraSky trial, a total of 83 patients were recruited (7 adolescents), randomized 1:1 to the satralizumab (41 patients) and to the placebo (42 patients) arms, with a median treatment duration of 107.4 weeks and 32.5 weeks, respectively. The primary endpoint was the first protocol-defined relapse in a time-to-event analysis. The major secondary endpoints were the change from the baseline to week 24 in the visual analogue scale (VAS) pain score and the Functional Assessment of Chronic Illness Therapy-Fatigue (FACITF) score. Relapse occurred in 20% in the satralizumab group and 43% in the placebo group; the percentages of patients free from relapse at 48 weeks was 89% and 66% in the satralizumab and placebo groups, respectively, and 78% and 59% at 96 weeks. In addition, the subgroup analysis revealed that 11% of AQP4-seropositive NMOSD patients (55 cases) in the satralizumab arm experienced relapses compared to 43% in the placebo group, while among 28 seronegative patients, relapse occurred in 36% and 43% in the satralizumab and placebo groups, respectively. Based on the subgroup analysis, it has been suggested that satralizumab reduces the risk of relapse compared to the placebo in AQP4-IgG-seropositive patients, but there was insufficient evidence to prove the agent’s effectivity in the seronegative participants. Regarding the secondary outcomes, no significant differences were found in either the VAS pain score or the FACIT-F score. Of note, in the satralizumab and the placebo arms, serious side effects occurred at similar percentages (17–21%). Injection-related reactions were more frequent in the satralizumab group (12% vs. 5%). 

In the SAkuraStar trial, patients were randomized 2:1 to the satralizumab monotherapy or placebo. A similar efficacy was demonstrated, with a significant reduction in the time to the first relapse and relapse risk in AQP4-IgG-seropositive NMOSD patients with an active disease. Relapse occurred in 30% of the satralizumab group and 50% of the placebo arm, with subgroup analysis showing 22% of seropositive patients treated with satralizumab relapsing compared to 57% in the placebo group. In the AQP4-seronegative subgroup, the percentages of relapse were 46% and 33%, respectively. No significant benefit was found on secondary outcome measures of pain or fatigue. Comparably to SAkuraSky, 19% of satralizumab-treated and 16% of placebo-treated NMOSD patients experienced adverse events, with injection reactions in 5% and 16%, respectively. In general, satralizumab showed a favorable safety profile in both studies, as no anaphylactic reactions, opportunistic infections, or deaths occurred. Only one patient in the SAkuraStar trial discontinued treatment due to pneumonia.

### 6.7. Emerging Therapeutic Strategies

A series of agents are currently under investigation for the prevention of disease activity in NMOSD. 

**Ublituximab** is a third-generation chimeric IgG1 monoclonal antibody with high affinity to the Fcy receptor IIIa (FCyRIIIA), an epitope on CD20-positive B-cells which is not targeted by rituximab, and a depleting larger number of B-cells [103,145]. Ublituximab allows for shortening the infusion duration and lowering doses compared to other anti- CD20 monoclonal antibodies and demonstrates enhanced antibody-dependent cellular cytotoxicity (ADCC) activity, while complement-dependent cytotoxicity (CDC) is retained [87,146]. In 2019, ublituximab was investigated in five AQP4-seropositive patients in a pilot safety study, phase Ib, as a novel add-on therapy in acute relapses of NMOSD (ON or TM) [92]. The agent was administered once in a 450 mg dose intravenously within 5 days of relapse onset as a concomitant treatment to high-dose intravenous corticosteroids (1000 mg per day on days 1–5). There were no severe adverse effects, and in three patients, EDSS improved at a 90 d follow-up. Two patients exhibited relapses within three months due to an insufficient depletion of B-cells [146].

Furthermore, **BAT4406F** is another potentially effective agent that is a fully humanized anti-CD20 monoclonal antibody to be investigated in a phase I RCT on safety, tolerability, and pharmacokinetics in NMOSD patients (NCT04146285). It will be administered via intravenous infusions, following an open-label dose escalation [37]. Additional potential B-cell-mediated therapeutic approaches that could be leveraged in the treatment of NMOSD include **chimeric antigen receptor (CAR) T cell therapy**, **belimumab** (an inhibitor of B lymphocyte stimulator (BLyS)), and several anti-CD20 monoclonal antibodies, such as **ocrelizumab**, **ofatumumab**, and **obinutuzumab** [104,147]. **Telitacicept** is a recombinant transmembrane activator and calcium modulator and cyclophilin ligand interactor fusion antibody acting by inhibiting both BLyS and proliferation-inducing ligands. In 2021, the agent was approved for the treatment of systemic lupus erythematosus, subcutaneously given weekly (160 mg), after showing efficacy and safety in a pivotal phase 2b trial (NCT02885610). An ongoing phase 3 randomized, placebo-controlled study is currently evaluating telitacicept in AQP4-ab-positive NMOSD patients without recent immunosuppressive treatment (NCT03330418, [107,148]). 

**Bortezomib** is a 26S proteasome inhibitor, FDA-approved for the treatment of multiple myeloma. The agent depletes plasma cells and is being evaluated in a range of autoantibody-driven neurologic autoimmune diseases, including myasthenia gravis and anti-NMDA-receptor encephalitis. Bortezomib was investigated in patients with highly relapsing NMOSD as an add-on medication in a small open-label study of five AQP4-Ab-positive Chinese women who had at least two relapses in the previous 6 months or three relapses throughout their life despite treatment with various immunosuppressants including prednisolone, azathioprine, rituximab, or cyclophosphamide (NCT02893111). The participants received four cycles of subcutaneous bortezomib at a dosage of 1 mg/m^2^ of body surface area on days 1, 4, 8, and 11 per cycle, followed by a 10-day treatment-free interval with concomitant oral steroid or azathioprine. Four out of five patients were relapse-free during a one-year follow-up. Side effects were mild and transient; however, long-term outcome and safety profiles were not reported. No patient experienced further neurological deterioration at the end of the study, and the median EDSS scores reduced from 5.5 at baseline to 3.5 after a 1-year follow-up, associated with an improvement in the pain scale. Furthermore, treatment significantly decreased serum AQP4 antibody titers, precursor B cell counts, peripheral blood CD19+ B cells, and mainly, CD138+ plasma cells. The findings suggest a promising role of bortezomib as an escalating approach in highly active NMOSD cases refractory to or intolerant of current immunosuppressants by depleting long-lived plasma cells. Phase 2 has been completed, but results are not yet available [87]. Importantly, the potentially unfavorable side effect profile of the agent is a matter of concern, as bortezomib is possibly associated with a rebound in plasma cell activity with an overshooting production of autoantibodies after cessation of the drug. In addition, it frequently induces peripheral neuropathy [103,149].

Subcutaneous injection of **batoclimab** (**HBM9161**) is being evaluated in NMOSD. It is a human monoclonal antibody that targets FcRn and accelerates the degradation of IgG, reducing total IgG levels in the blood (including pathological IgG). Based on its suggested anti-lgG properties, it is expected to rapidly reduce AQP4-IgG levels when administered with IVMP. The agent is injected subcutaneously at a dose of 340 mg or 680 mg weekly for a period of 4 weeks, and is being evaluated in a phase 1, open-label dose exploration study of NMOSD patients experiencing relapses (NCT04227470). Furthermore, a new study comparing thw efficacy and safety of immunoadsorption and PLEX for acute relapses of refractory NMOSD (CAMPUS; NCT04064944) has been announced, but is not recruiting yet [87].

**Imlifidase**, an IgG-degrading bacterial enzyme, is another agent that could be effective in AQP4-seropositive NMOSD. It mediates the cleavage of IgG molecules into Fab and Fc segments. The concept was conceived based on promising results from animal models of NMOSD, which have demonstrated that the transformation of AQP4 antibodies into inactive antibodies by the microbial-mediated deglycosylation of IgG heavy chains may have a role in NMOSD therapeutic armamentarium. Leveraging the same strategy of downregulating pathogenic autoantibodies, the potential effectivity of **rozanolixizumab** and **efgartigimod** could be suggested. These agents constitute inhibitors of neonatal Fc receptors (FcRn), crucial for antibody stability [150].

Bruton’s tyrosine kinase (BTK) is an enzyme that plays a crucial role both in B cell development by transmitting intracellular signals from the pre-B cell receptor, and in the Fc-receptor-mediated activation of myeloid cells. It promotes antigen recognition via antibody-mediated opsonization. In contrast to the typical CD20 monoclonal antibodies, BTK inhibitors inactivate B cells without causing prolonged and repeated B cell depletion, thus lowering the risk for serious opportunistic infections. BTK inhibitors are being developed as therapeutic agents for MS, with promising findings from phase 2 clinical trials, while phase 3 trials are underway. An open-label phase 2 trial will be starting soon to evaluate the efficacy and safety of the agent **SHR1459 (Bruton’s Tyrosine Kinase Inhibitor)**, orally administered, in preventing relapses in NMOSD (NCT04670770) [87,151]. 

**Bevacizumab** is an anti-angiogenic compound, and more specifically, a monoclonal immunoglobulin that targets vascular endothelial growth factor (VEGF), that has been widely used for the treatment of retinal diseases. VEGF-neutralizing antibodies such as bevacizumab have the potential to restore BBB integrity, as antibodies targeting brain microvascular endothelial cells (BMEC) are believed to induce disruption of the BBB mediated by VEGF, leading to pathogenic AQP4 antibodies entering into the central nervous system. Of note, anti-BMEC antibodies were found in the sera of 10/14 NMO patients, but were absent in MS and healthy controls [102,152]. Data on its efficacy as an add-on agent for treatment of ON and/or TM in NMOSD come from a phase 1b trial where bevacizumab proved to be effective and safe in 10 patients, with none requiring escalation to PLEX after high-dose IVMP plus IV bevacizumab. The suggested approach is the infusion of 10 mg/kg intravenously at the onset of exacerbation and, if needed, a subsequent dose during the plasma exchange phase [107].

**Ravulizumab** is a second-generation monoclonal antibody targeting C5 and blocking its activation, thus inhibiting C5 cleavaging into fragments C5a and C5b. It is derived from eculizumab and was designed to provide prolonged therapy intervals by utilizing the “Ab-recycling” approach. The agents show increased affinity for the neonatal receptor FcRn, and rapid endosomal dissociation of the ravulizumab-C5 complex allows lysosomal degradation of C5 while recycling ravulizumab to the vascular space through the FcRn 66. Ravulizumab has an extended serum half-life (3 to 4 folds) compared to its predecessor. Based on evidence of non-inferiority to eculizumab derived from two large phase III trials in patients with paroxysmal nocturnal hemoglobinuria, the agent was approved by the FDA and EMA for use in adult patients. Ravulizumab is administered every 2 months. Since December 2019, a phase 3, external, placebo-controlled, open-label, multicenter study of ravulizumab efficacy and safety in AQP4-Ab-positive NMOSD patients has been underway (NCT04201262) [153,154,155]. 

Alternative ways to target the complement cascade are being explored based on evidence that blockage of the C1 component prevents the formation of proinflammatory anaphylatoxins C3a and C3b while preserving the lectin pathway, which is important for neutralizing encapsulated bacteria. Indeed, in animal models, **C1qmab**, a monoclonal antibody against C1q components, effectively reduced complement-dependent cytotoxicity. In addition, an open label, phase 1b trial which investigated the **C1 esterase inhibitor** as a concomitant therapy to steroids for the management of acute NMOSD relapses showed favorable results regarding safety and effectivity, with 90% of patients returning to their baseline EDSS score [102]. 

Granulocyte-targeting strategies have also been considered in NMOSD treatment, as animal models suggest that granulocytes mediate NMO pathogenesis, and neutrophils and eosinophils are highly prevalent in NMOSD lesions. It is suggested that neutrophil entry into the CNS is an early step in the formation of NMOSD lesions. Blocking neutrophil elastase, a proteolytic, highly destructive enzyme that triggers the production of inflammatory cytokines, helps reduce neutrophil entrance into the brain. **Sivelestat** is a neutrophil elastase inhibitor which is being investigated in acute NMO relapses. In a mouse model of experimental autoimmune encephalomyelitis, the agent reduced ADCC. Phase I/II clinical trials were discontinued for various reasons. Sivelestat has already been approved in Japan and Korea for ARDS treatment [156].

**NPB-01 IVIg** (400 mg/kg/day for 5 consecutive days) is being investigated for its potential role in NMOSD mediated by the inactivation of auto-reactive T-cells. However, a phase 2 RCT in AQP4-ab-positive NMOSD patients did not improve responses when added to IVMP, but detailed results are not available. 

Cetirizine, a second-generation H1 antagonist that stabilizes eosinophil degranulation, was investigated in a small open label add-on pilot study and showed a decrease in ARR in NMO patients at a 1-year follow-up; however, no significant difference in EDSS scores were observed [157]. Cetirizine was administered orally at 10 mg each day. Another potential consideration for use in NMOSD is anti-IL-5 agents, which deplete eosinophils [158].

**Aquaporumab** is a recombinant monoclonal antibody derived from clonally expanded mouse CSF plasma cells with a point mutation in the area that codes for effector Fc IgG functioning. The agent constitutes a targeted non-immunosuppressive therapy that binds AQP4 with high-affinity cells, displacing AQP4-Ab from binding. The Fc portion of aquaporumab specifically aims at disabling AQP4-Ab from triggering CDC or ADCC downstream mechanisms. A study in a mouse model of NMO has showed that aquaporumab prevented the formation of new NMO lesions through steric competition with pathologic AQP4 antibodies [159]. Recently, Duan et al. described **AQmab**, which has an eightfold increased binding affinity to the AQP4 receptor compared to aquaporumab [147]. 

Another idea worth noting would be the induction of **immune tolerance** to the autoantigen by vaccination, as the majority of NMOSD patients have underlying AQP4 autoimmunity with the autoantigen clearly defined. 

### 6.8. Cell-Based Therapies

Cell-based therapies are gaining momentum as promising treatments in the armamentarium against severe autoimmune diseases, such as refractory NMOSD and MOGAD, aiming at either the depletion of autoreactive effector cells or the modulation of autoreactive T and B cell responses, resulting in the restoration of tolerance. Various cellular treatment approaches have been investigated in NMOSD and occasionally in MOGAD as well, including autologous hematopoietic stem cell transplantation (HSCT) and chimeric antigen receptors (CAR)-T cell, tolerogenic dendritic cell, and mesenchymal stem cell treatment. The therapies have entered early-stage clinical trials or have been used as a rescue treatment in treatment-refractory or highly aggressive cases. Progress in the field is slowed down by the rarity of the diseases, the shortage of biomarkers able to predict long term outcomes and effectiveness, challenges in the manufacturing of cellular products, and the lack of adequate animal models that mirror the human disease [160]. 

**Hematopoietic Stem Cell Transplantation (HSCT)** in NMOSD and MOGAD is aimed at achieving the elimination of the dysfunctional immune system with high-dose chemotherapy and rebuilding through hematopoietic stem cell infusion in order for long-term remission to be achieved. **Autologous Stem Cell Transplantation (AHSCT)** is preferred as it avoids graft-versus-host reactions. Complications of AHSCT include neutropenic fever, serious infections, electrolyte abnormalities, blood pressure fluctuations, and the emergence of new autoimmune diseases, including myasthenia gravis and hyperthyroidism. Mortality associated with the therapy has improved significantly over the last several decades and is now around 0.2% [160]. The first case report of an autologous stem cell transplantation in a 23-year-old severely affected patient with refractory NMOSD was published in 2010. In a 12-month follow-up, the patient remained blind, but paraparesis and dysesthesia remitted [161]. 

Results of the two largest studies of AHSCT in NMOSD patients were discrepant, possibly due to the choice of conditioning regimen [162,163]. The European Registry retrospective AHSCT study included 16 patients with NMOSD refractory to immunosuppressants, with 10% remaining relapse-free and 48% with progression-free survival at 5 years, but the study did not use rituximab. Eighty percent of initially seropositive patients remained seropositive throughout the study. In contrast, in a US-based clinical trial that included 13 patients (11 AQP4-IgG-seropositive and 1 with neuropsychiatric SLE), all participants followed the same therapeutic approach treatment, consisting of cyclophosphamide, rituximab, anti-thymocyte globulin, and plasmapheresis. Eighty-three percent of patients were relapse free at 5 years off all immunosuppressants. Furthermore, at 1 and 5 years after transplant, improved scores in the EDSS and in the Neurological Rating scale were recorded. Interestingly and importantly, 9 of 11 AQP4-seropositive patients in the US study seroconverted to being AQP4-seronegative after HSCT, and all of them remained relapse-free at the last follow-up despite the fact that two regained AQP4-seropositive status within 2 years of transplant. The two patients who remained AQP4-seropositive throughout the study were the ones who had clinical relapses. In addition, complement-activating and cell-killing ability was lost in six of seven patients. The possibility of prolonged drug-free remission with conversion to AQP4-IgG seronegativity following nonmyeloablative hematopoietic stem cell transplantation warrants further study. Each study recorded one death (patient with coexisting SLE in the US trial due to SLE complications) [162,163]. 

A recent meta-analysis in 2020 including the aforementioned three studies evaluated 31 NMOSD patients in total who underwent AHSCT [164]. Cumulative progression-free survival was 76% during a follow-up period between 2 and 13 years. Treatment-related mortality was 0%. Despite the promising results, a number of patients had persisting AQP4 antibodies and relapsed within 5, and the optimal conditioning regimen remains to be determined as well [110] Based on these findings, the European Bone Marrow Transplantation (EBMT) Autoimmune Diseases Working Party (ADWP) issued guidelines recommending the use of AHSCT in NMOSD as a clinical option, with grade II evidence, in therapy-refractory patients [104]. 

Only a few patients treated with **allogeneic HSCT (alloHSCT)** have been reported [105,165]. AlloHSCT has the potential for a more profound immunotherapeutic effect, eliminating all autoreactive lymphocytes by allogeneic donor T lymphocytes. In addition to the increased risk of morbidity and mortality after alloHSCT, there are reports of the development of immune-mediated peripheral and central nervous system diseases, including a case of MOGAD after alloHSCT in haematological patients [105,165]. Due to limited clinical evidence, alloHSCT in NMO was classified as developmental by the EBMT-ADWP and is currently not recommended as a clinical option [104].

One phase Ib, open-label, multiple-ascending-doses, single-center clinical trial was conducted recently in Spain, evaluating the efficacy and safety autologous of **tolerogenic peptide-loaded dendritic cells (DC)** in 4 AQP4+ NMOSD patients [166]. The tolerogenic phenotype of DC was induced by the addition of dexamethasone; DC from NMOSD patients were stimulated with seven myelin peptides and AQP4. Three doses of tolerogenic DC were administered intravenously at week 0, 2, and 4 at progressively increasing doses, and all patients received concomitant treatment with rituximab [3] or mycophenolate [1]. All patients remained clinically stable, no relapses occurred, and the tolerogenic DC-based therapy proved to be safe. Immunological analysis demonstrated a trend of decreased T cell proliferation, a significant increase in Interleukin-10 production, and an upregulation of type 1 regulatory T (Tr1) cells, findings confirmatory of tolerance induction [166].

Another cell-based therapeutic approach is the employment of **chimeric antigen receptors (CAR)**, proteins carrying both an antigen-binding and a T-cell-activating function, allowing T cells to target a specific protein. B cell targeting using **CAR-T cell** therapy is being investigated mainly in the treatment of hematological malignancies, but there is also an emerging interest in the field of autoimmunity, where dysregulated B cell activation leads to an antibody-mediated targeting of healthy body tissue. Breaking the immune tolerance towards autoreactive immune cells induces the cytotoxic death of these specific cells, which may downregulate the immune overactivation driving autoimmunity. Indeed, recent promising results come from a murine model of SLE [160]. In the field of NMOSD, an open-label phase I clinical trial is currently underway, utilizing B cell maturation antigen **(BCMA) CAR-T cell** therapy in patients with refractory AQP4-IgG-seropositive NMOSD (ClinicalTrials.gov NCT04561557). Twelve NMOSD patients will be enrolled and receive BCMA CAR-T cells following lymphodepletion with cyclophosphamide and fludarabine. Primary outcome measures include the incidence of dose-limiting toxicities and adverse events. The concentration of AQP4-IgG titers in the serum 3 months after infusion and the CAR-T cell proliferation 2 years after infusion will be studied as secondary outcome measures, together with clinical and radiological outcomes, including the annualized relapse rate and active MRI lesions. The first results of this clinical trial are expected by the end of 2023 [160].

**Mesenchymal stem cells** (MSCs) constitute multipotent stromal progenitor cells, derived from allogeneic human-umbilical-cord-derived tissue (hUC-MSC), autologous bone marrow (bMSC), or autologous adipose tissue. Among the beneficial effects of MSC treatment are their regenerative potential, immunomodulatory properties inhibiting pro-inflammatory cytokines, and a neuroprotective action by the secretion of neurotrophic and survival-promoting growth factors. In the field of NMOSD, clinical trials with both bMSC and hUC-MSC have been conducted. Compared to bMSC, hUC-MSC are easily collectable, and although not autologous, these MSCs have a low risk for the induction of allogeneic immune responses and consequently transplant rejection [106,118]. In a pilot study, 15 AQP4-seropositive NMOSD patients were treated with a single intravenous infusion of autologous bMSC, and at 2 years follow-up, favorable results were observed regarding relapses (87% relapse-free) and disability (improvement in 40%). HUC-MSC in the treatment of AQP4 IgG+ NMOSD patients was first investigated in 2012 in five cases, when the cells were administered by an intravenous and intrathecal route combined, divided over four infusions; favorable results were found following transplantation in terms of relapses, EDSS score, and peripheral blood B lymphocyte counts. Interestingly, the 10-year follow up in 2020 demonstrated that four out of five treated NMOSD patients showed reduced annual relapse occurrence compared to before treatment, with only two patients completing the 10-year follow-up period due to the death of two patients (attributed to rapid disease progression) and failure to follow up with one patient. The safety profile was promising, with no observed long-term tumor formation or peripheral organ disorders. The investigators concluded that hUC-MSC transplantation warrants further clinical trials [160,167].

### 6.9. Remyelination

Therapeutic approaches aimed at improving regeneration and restoring functionality are still missing. A future treatment pathway inducing remyelination or myelin repair would be beneficial. Promoting the differentiation and proliferation of oligodendrocyte precursor cells (OPC) to mature oligodendrocytes capable of myelination might be a key component of the concept. **Clobetasol** has been shown to promote OPC differentiation in cultured cells and to induce remyelination in mouse brains with AQP-IgG and complement-induced injury [168]. 

## 7. Therapeutic Approach to AQP4-IgG-Seronegative NMOSD Patients

Although AQP4-ab-negative patients are considered in the 2015 NMOSD diagnostic criteria, a large diagnostic disagreement has been reported in this subgroup of patients, even among experts in this field, owing to the inconsistent use of the criteria. Consequently, the diagnosis of patients who fulfil the 2015 diagnostic criteria for AQP4-IgG seronegative NMOSD patients requires caution, and seronegative status should be confirmed with cell-based assays, with repeat blood work at least two to three times in a period of 6–9 months. In addition, AQP4-IgG-seronegative patients should be assayed for MOG-IgG by cell-based assays. 

There are therapeutic challenges for double-seronegative NMOSD patients (seronegative for both AQP4-IgG and MOG-IgG), as the recent randomized placebo-controlled clinical trials of eculizumab, inebulizumab, and satralizumab either did not include such patients (eculizumab) or failed to provide evidence that the newer agents are effective for relapse prevention (inebulizumab and satralizumab) in this group. In the N-MOmentum and SAkuraStar trials, which compared a placebo to inebilizumab and satralizumab, respectively, AQP4-seronegative patients were included, but these trials were not sufficiently powered to evaluate the response in this subgroup, rendering the results primarily applicable to AQP4-seropositive patients. 

Recently, the Spanish NMO Study Group reported that double-seronegative and AQP4-IgG-seropositive NMOSD patients had a similar clinical outcome, while those seropositive for MOG-IgG had a more favorable prognosis [169]. Moreover, a study in France that included 67 patient and employed MMF as a first-line therapy concluded that the agent was effective in relapse prevention and disability stabilization/improvement in NMOSD patients (based on 2015 diagnostic criteria), irrespective of the seropositivity status (AQP4-IgG seropositive, MOG-IgG seropositive, or double-seronegative) [113]. In addition, another recent multicenter retrospective study of 245 NMOSD patients found a similar efficacy of rituximab and MMF both in AQP4-IgG-seropositive and double-seronegative NMOSD patients [170].

### 7.1. MOG

The myelin oligodendrocyte glycoprotein is one of several proteins produced by oligodendrocytes, the myelin-forming cells of the CNS. Together with other proteins such as myelin basic protein (MBP), proteolipid protein (PLP), and myelin-associated glycoprotein (MAG), MOG is an essential component of oligodendrocyte surface membranes. These glycoproteins have fundamental roles in the formation, maintenance, and disintegration of myelin sheaths [171]. Compared to other glycoproteins MOG is only found in relatively small amounts within myelin; however, its structure (extracellular IgV domain) and the outmost external location on myelin sheaths make it easily accessible to the potential antibodies and T-cell response involvement. MOG expression starts when myelination begins and is thus a possible differentiation marker for oligodendrocyte maturation. Several essential functions of MOG are suggested: the regulation of oligodendrocyte microtubule stability, maintaining the structural integrity of the myelin sheath by its adhesion features, and the mediation of interactions between myelin and the immune system [172]. In humans and rodents, the MOG gene is located in the major histocompatibility complex (MHC) locus. Molecules encoded by this region are found on the surfaces of cells and are involved in antigen presentation, inflammation regulation, the complement system, and the innate and adaptive immune responses. In addition, the gene has a certain structural similarity to the B7-CD28 superfamily—encoded proteins are expressed on the surface of antigen-presenting cells (APC) [173]. In addition, MOG can directly activate the classical pathway of the complement cascade; reports from experimental studies suggest that the binding of MOG to the C1q and C3d components can activate the complement system. 

Neuropathological evidence has shown that the inflammatory infiltration in MOGAD consists mainly of CD4+ T cells and granulocytes, in contrast to MS, where CD8+ T cells predominate. Compared to MS, intracortical rather than leukocortical demyelinated lesions were more common. Importantly, AQP-4 was preserved, as MOGAD is not an astrocytopathy. Complement deposition within active lesions was observed, but not on astrocytes or glia limitans. Contrary to expectations, MOG was not preferentially lost [174]. 

NMOSD and MOGAD are two antibody-mediated entities; however, both have different targets. AQP4-ab-positive NMOSD is characterized by AQP4 loss, dystrophic astrocytes, and the absence of cortical demyelination [87]. By contrast, MOGAD pathology is characterized by the coexistence of perivenous and confluent primary demyelination, with partial axonal preservation and reactive gliosis in the white and gray matter, and with a particular abundance of intracortical demyelinating lesions [174]. This occurs on the background of CD4-dominated T cells and granulocytic inflammatory infiltrates [87]. In addition, contrary to classical AQP4-ab-positive NMOSD, in MOGAD, the expression of AQP4 is preserved [174].

The phenotype of MOGAD is broad and includes ON, TM, and acute demyelinating encephalomyelitis (ADEM). ON is the most common presentation in adults, whereas ADEM is in children [175]. In MOGAD, disability appears to depend on relapses, with severe disability being reported in 47% of adult MOGAD patients, in >70% of whom it results from the first attack [176]. Clinical characteristics suggestive of MOGAD-ON include recurrent ON, bilateral involvement, prominent disc edema, and longitudinally extensive ON and/or perineural enhancement of the optic nerve on MRI [96]. Although the nadir of vision loss is severe with MOGAD-ON, the recovery is typically better than with AQP4-IgG ON, and, in general, MOGAD-associated demyelination has been suggested to have a more favorable prognosis compared with AQP4-seropositive NMOSD, featuring a lower EDSS and reduced risk of visual and motor disability. The clinical course of MOGAD can be monophasic; however, approximately 50% of patients with MOGAD will experience a recurrent demyelinating attack, most commonly ON [177]. 

Disability from both AQP4- and MOG-associated ON is accumulated by poor recovery from attacks. Interestingly, when serum samples from 177 of 448 patients enrolled in the ONTT were assayed for AQP4- and MOG-IgG, only four MOG-IgG-seropositive patients were identified. Therefore, the results of the ONTT are not informative regarding the impact of high-dose corticosteroids on visual recovery in NMOSD-ON and MOG-ON. Importantly, the clinical course of MOGAD-ON, as in NMOSD-ON, differs from idiopathic and MS-associated ON (where steroids do not affect the ultimate visual outcome) by being typically briskly steroid-responsive and sometimes steroid-dependent [38].

Treatment with corticosteroids is almost always used in acute MOGAD-ON to aid in visual recovery, and there are limited data on the natural history without treatment. As such, new onset diseases or acute relapses are typically treated with high-dose IV methylprednisolone for 3–5 days. According to a European cohort, a number of patients had an extremely rapid return to baseline within 48 h following steroid initiation [175]. 

Acute attacks that respond poorly to steroids can be treated with PLEX or immunoadsorption. Observational studies have shown that, similarly to NMOSD, a shorter time to treatment correlated with less retinal nerve fiber layer losses and better visual outcomes [36]. Similarly to NMOSD, “time equals vision”. The optimal treatment initiation may be by day 4, but treatment even before day 7 still offers an opportunity for very good visual outcomes in MOGAD [36]. 

Typically, MOGAD-ON neuritis relapses respond well to steroids, but patients are often vulnerable to relapses on tapering or withdrawal of steroids [177]. A recurrent course is associated with higher titers of MOG-IgG during the first months and/or maintenance of seropositive status despite treatment [175]. In contrast, low titers or seroconversion to negativity in the early course represent a reliable predictor of a monophasic course. 

The treatment of MOGAD has been largely extrapolated from AQP4-IgG NMOSD and is currently understandardized, still based on clinical experience and observational studies (Class IV evidence), with no approved drugs, to date, for long-term relapse prevention in adult patients. No phase III multicenter randomized clinical trials have been performed to assess treatment effectiveness in MOGAD, due to difficulties related to the recent recognition and low prevalence of this disease, the wide age range, and broad clinical spectrum. MOGAD-ON frequently recurs when patients are on no-maintenance long-term treatment, with 80% of patients having two or more attacks over a median time of 2.9 years [176]. Prior retrospective studies suggest that long-term immunosuppressant therapy may reduce the frequency of recurrent attacks, while most DMTs used to treat MS have not demonstrated usefulness in preventing relapses in MOGAD. Compared to pediatric patients, adult MOGAD patients may have a higher risk of relapses and a worse functional recovery as well as a shorter median time until a second attack, supporting the use of long-term relapse prevention treatments in adult seropositive patients with ON and/or TM. 

**Maintenance oral steroids** at the lowest possible dose are an effective treatment strategy in MOGAD. The Australasian and New Zealand MOG Study Group recently showed that relapses commonly occurred with doses of <20 mg prednisone per day in adults, and that a duration of treatment or less than 3 months was associated with a 2-fold higher risk of relapses, compared to patients treated for a longer time [175]. In addition, the concomitant use of oral steroids as an adjunct to immunosuppressive drugs was accompanied b ay reduced risk for relapses (5% vs. 38% on immunosuppressive monotherapy). Some patients on maintenance low-dose prednisone alone had a relapse-free course, indicating the efficacy of steroids in sustaining remission, but the significant long-term metabolic and bone health-related adverse effects warrant caution. Interestingly, a subgroup of MOGAD patients remained relapse free on no immunotherapy for a long time after the initial treatment with steroids, to only experience a relapse after many years [175]. Another group in China demonstrated that the early tapering or discontinuation of oral steroids within 30 days had as an outcome a relapse in 59% of patients [178]. In conclusion, a prolonged steroid taper may reduce the chance of early relapses and provide an acceptable maintenance option, with close monitoring during and after steroid cessation. 

Based on data from a recent, large multicenter cohort of MOG-IgG-positive patients conducted by Mayo Clinic, **maintenance IVIg**, at 3- or 4-week intervals, applied in 10 patients (5 pediatrics), demonstrated the lowest relapse rate (only 20% had a relapse) compared to alternative immunosuppressives with a relapse rate >50% (59% for AZA, 73% for MMF, 62% for RTX) [177]. Chen et al. suggested that IVIg effectivity in suppressing future attacks was independent of a bias toward using IVIg in patients with a more benign disease, and that long-term IVIg is an effective maintenance immunotherapy for patients with MOGAD [123]. Previous small retrospective studies support these results, especially in children [126]. By contrast, the Australian cohort showed a higher relapse rate in three out of seven patients receiving long-term IVIg; however, the median ARR for the cohort was 0, and the highest relapse rate in patients treated with IVIg was the lowest among the treatments evaluated (range 0–0.75) [175,177]. Furthermore, IVIg treatment efficacy was shown in a large European retrospective cohort of MOG-IgG-positive patients with ON and/or TM as a therapeutic approach after an acute relapse showing favorable results, as 50% of patients experienced complete (or almost complete) recovery and 44% partial recovery (measured by visual acuity and EDSS) [175].

**Azathioprine** and **MMF** seem to be effective and safe therapeutic strategies for long-term immunosuppression in adult MOGAD patients, with failure and intolerance being the most frequent causes for the agents’ discontinuation. The agents can be used as a monotherapy or in combination with oral steroids. 

Based on a systematic review that included 17 articles, azathioprine (2–3 mg/kg/day divided into 2–3 doses) achieves a reduction in the mean and median ARR, as well as the stabilization or improvement of the EDSS [179]. Azathioprine was found to have the second lowest post-treatment ARR after IVIG, although the slightly lower pretreatment ARR for recipients of azathioprine compared to patients receiving the other therapies could have led to a bias. Patients on AZA were also more frequently on concomitant maintenance prednisone. The interval between the initiation of azathioprine and the first relapse ranged from 3 to 9 months (median of 6 months) [179,180]. 

Cobo-Calvo et al. recently reported a significant reduction in relapses in patients treated with MMF in a cohort of Spanish and French adult patients with relapsing MOGAD [128]. Furthermore, the systematic analysis in adult MOGAD patients, showed the efficacy of MMF (1500–3000 mg/day divided into two doses) in a total sample of 96 treated patients with the agent, regarding ARR and EDSS indices [179]. Similar promising results come from a more recent prospective study, especially in a subset of patients with isolated ON or high MOG-IgG titers. Eighty-six percent of patients had a reduced risk for relapse after 400 days of follow up [181]. In the Australian cohort, MMF also appeared to be effective, but treatment failure rates were higher, and relapses were often associated with steroid tapering, suggesting the steroid was producing the benefit in these patients [175]. Consistent findings were derived from the Mayo Clinic as well, where MMF use in 13 patients documented a more modest reduction in relapse rates compared to the other immunosuppressive agents [177].

Limited data about the potential use of cyclophosphamide in MOGAD are available. Similar to the Australian cohort, which reported 50% failure [177], in the Mayo Clinic cohort, two of three patients had relapses during treatment with the agent [177]. Chen et al. commented that even though the lack of apparent efficacy could be attributed to the small total number of patients treated with IV cyclophosphamide or to the reservation of this potent agent for the most severe and refractory cases, the findings may indicate that cytotoxic CD8 T cells are not key effectors of MOGAD pathogenesis. Confirmatory of the potential lack of the agent’s efficacy in MOGAD patients is the fact that when the two patients who relapsed early on cyclophosphamide switched to rituximab, they stabilized without further relapse [177].

Whittam et al. showed in a multicenter study of 98 patients treated with rituximab that the agent reduces the relapse rate for MOGAD, but the benefit did not appear to be as great as for AQP4-IgG-positive NMOSD [182]. Recently, the same group demonstrated in a study of 71 adult MOGAD patients on rituximab a relapse rate of 42%, with a median follow-up time of 12.7 months [183]. Interestingly, the investigators found that MOG-specific B cells were only detected in about 60% of these patients, indicating that MOG-specific B cells are not linked to levels of serum MOG-Abs, casting doubt on whether B-cell-depleting treatments should be used in MOG-seropositive patients. The findings of the aforementioned studies concur with the data derived from the systematic review, which concluded that, similar to AQP4-ab-positive NMOSD patients, new relapses within the few weeks after the first rituximab infusion occurred in about 30% of MOGAD patients despite B cell depletion, with a median time from the most recent infusion to the first relapse of 2.6 (range: 0.6–5.8) months [183]. It has been also suggested, that in relapsing MOG-seropositive patients needing rituximab, regular CD19 monitoring and proactively redosing a brittle patient in the event of B-cell repopulation might reduce the incidence of repopulation relapses, as this has been demonstrated in NMOSD [175]. 

Emerging therapeutic approaches which have been used successfully in the treatment of NMOSD could also be evaluated in MOGAD. To date, tocilizumab has been used with varied effectivity in some patients with rituximab-refractory MOGAD. Furthermore, the NMOmetum trial, which compared inebelizumab vs. placebo administration in NMOSD patients, also enrolled seven adult MOGAD patients, but separate outcomes for the MOGAD subgroup were not specifically reported [184]. Novel, future, potential treatments, also being investigated in NMOSD include: efgartigimod, a synthetic IgG1 Fc analog, which has shown efficacy as a substitute for IVIG in treating the IgG-mediated neuromuscular disorder myasthenia gravis; rozalixizumab, an inhibitor of the neonatal Fc receptor; and Bruton′s tyrosine kinase inhibitors.

### 7.2. Chronic Relapsing Inflammatory Optic Neuropathy

The main characteristic of patients with chronic relapsing inflammatory optic neuropathy (CRION) is the rapid and excellent response to corticosteroid therapy, as well steroid dependence, with relapses within weeks or months after the withdrawal of or a decrease in corticosteroids [185]. CRION requires careful consideration and differentiation from typical, demyelinating optic neuritis, since the treatment is entirely different, and the outcome without treatment is likely to be very poor. Furthermore, the standard treatment of typical ON is not adequate for CRION [186]. 

Already when CRION was first described by Kidd in 2003, the authors concluded that treatment with corticosteroids was able to induce an abrupt and prompt relief of pain and, at times, a complete restoration of normal visual acuity and color vision even months after the onset of symptoms [186]. There was evidence that following steroid withdrawal, patients tended to relapse, necessitating long-term immunosuppression, which appears to arrest progression of the disease in the majority of cases [186]. Since then, the clinical experience has evolved, and although CRION patients generally tend to respond well to steroids, cumulative damage can lead to poor visual outcomes and structural changes in RNFL and GCL complexes permanently. Indeed, a recent study showed that up to 25% of patients can end up with a final visual acuity <20/40 [187]. Consequently, early diagnosis and timely management are key for restoring as well as preserving vision. 

For the time being, treatment recommendations are based on the activity of the disease and the clinical experience with related disorders, as no CRION-specific formal guidelines have been established yet. The general approach in the acute phase of the disease is the administration of IV methylprednisolone 1 mg/kg for 3–5 days, possibly with added IVIg or PLEX in severe cases, followed by oral steroids (1 mg/kg) with gradual tapering, as an abrupt withdrawal of treatment may lead to the irreversible worsening of visual acuity [185,188]. Given that relapses are common, the abrupt disruption of treatment should be avoided, and the minimal effective glucocorticoid dose be identified. In addition, the early initiation of a steroid-sparing immunomodulatory agent would be a reasonable consideration given the well-known iatrogenic morbidity of glucocorticoids. Azathioprine, rituximab, IVIg, cyclophosphamide, and methotrexate have been used for long-term and short-term treatment in single reports [136]. Natalizumab has also been employed [185]. Of course, if MOG or AQP4 seropositivity or another connective tissue disease can be identified, the therapeutic approach should be targeted at those disorders [188]. 

## 8. Conclusions

Typical and atypical ON follow a different natural history, rendering crucial the timely differentiation between them. Treating typical ON primarily accelerates recovery without any effect on the final visual outcome. However, the diagnosis of CIS such as ON provides the opportunity to closely monitor a patient clinically, and consider early initiation of MS DMTs. However, more work is needed to find remyelinating and reparative treatment approaches.

Among atypical causes of ON, NMOSD-related ON stands out in its severity. Prompt and aggressive treatment is needed to save vision and CNS functioning. In addition, the early initiation of relapse-prevention strategies is recommended. Slow steroid tapering and the recruitment of additional long-term therapies should be considered for MOGAD-ON and CRION.

## References

[1] Menon V., Saxena R., Misra R., Phuljhele S. (2011). Management of optic neuritis. Indian J. Ophthalmol..

[2] Morrow S.A., Fraser J.A., Day C., Bowman D., Rosehart H., Kremenchutzky M., Nicolle M. (2018). Effect of treating acute optic neuritis with bioequivalent oral vs intravenous corticosteroids—A randomized clinical trial. JAMA Neurol..

[3] Huang W.J., Chen W.W., Zhang X. (2017). Multiple sclerosis: Pathology, diagnosis and treatments. Exp. Ther. Med..

[4] Compston A., Coles A. (2008). Multiple sclerosis. Lancet.

[5] Wingerchuk D., Lucchinetti C., Noseworthy J. (2001). Multiple Sclerosis: Current Pathophysiological Concepts. Lab. Investig..

[6] Lucchinetti C., Brück W., Parisi J., Scheithauer B., Rodriguez M., Lassmann H. (2000). Heterogeneity of multiple sclerosis lesions: Implications for the pathogenesis of demyelination. Ann. Neurol..

[7] Traugott U., Reinherz E.L., Raine C.S. (1983). Multiple sclerosis. Distribution of T cells, T cell subsets and Ia-positive macrophages in lesions of different ages. J. Neuroimmunol..

[8] Ferguson B., Matyszak M.K., Esiri M.M., Perry V.H. (1997). Axonal damage in acute multiple sclerosis lesions. Brain.

[9] Bitsch A., Schuchardt J., Bunkowski S., Kuhlmann T., Brück W. (2000). Acute axonal injury in multiple sclerosis. Correlation with demyelination and inflammation. Brain.

[10] Hu D., Ikizawa K., Lu L., Sanchirico M.E., Shinohara M.L., Cantor H. (2004). Analysis of regulatory CD8 T cells in Qa-1-deficient mice. Nat. Immunol..

[11] Toosy A.T., Mason D.F., Miller D.H. (2014). Optic neuritis. Lancet Neurol..

[12] Babbe H., Roers A., Waisman A., Lassmann H., Goebels N., Hohlfeld R., Friese M., Schröder R., Deckert M., Schmidt S. (2000). Clonal expansions of CD8(+) T cells dominate the T cell infiltrate in active multiple sclerosis lesions as shown by micromanipulation and single cell polymerase chain reaction. J. Exp. Med..

[13] Carlström K.E., Zhu K., Ewing E., Krabbendam I.E., Harris R.A., Falcão A.M., Jagodic M., Castelo-Branco G., Piehl F. (2020). Gsta4 controls apoptosis of differentiating adult oligodendrocytes during homeostasis and remyelination via the mitochondria-associated Fas-Casp8-Bid-axis. Nat. Commun..

[14] Chamberlain K.A., Chapey K.S., Nanescu S.E., Huang J.K. (2017). Creatine enhances mitochondrial-mediated oligodendrocyte survival after demyelinating injury. J. Neurosci..

[15] Jin J., Smith M.D., Kersbergen C.J., Kam T.-I., Viswanathan M., Martin K., Dawson T.M., Dawson V.L., Zack D.J., Whartenby K. (2019). Glial pathology and retinal neurotoxicity in the anterior visual pathway in experimental autoimmune encephalomyelitis. Acta Neuropathol. Commun..

[16] Cannella B., Raine C.S. (1995). The adhesion molecule and cytokine profile of multiple sclerosis lesions. Ann. Neurol..

[17] Kuhlmann T., Miron V., Cui Q., Wegner C., Antel J., Brück W. (2008). Differentiation block of oligodendroglial progenitor cells as a cause for remyelination failure in chronic multiple sclerosis. Brain.

[18] Goodkin D.E., Rudick R.A., Goodkin D.E. (1992). The Natural History of Multiple Sclerosis. Treatment of Multiple Sclerosis.

[19] Koopmans R.A., Li D.K.B., Oger J.J.F., Kastrukoff L.F., Jardine C., Costley L., Hall S., Grochowski E.W., Paty D.W. (1989). Chronic progressive multiple sclerosis: Serial magnetic resonance brain imaging over six months. Ann. Neurol..

[20] Prineas J.W., Barnard R.O., Kwon E.E., Sharer L.R., Cho E.S. (1993). Multiple sclerosis: Remyelination of nascent lesions. Ann. Neurol..

[21] Lotan I., Hellmann M.A., Benninger F., Stiebel-Kalish H., Steiner I. (2018). Recurrent optic neuritis—Different patterns in multiple sclerosis, neuromyelitis optica spectrum disorders and MOG-antibody disease. J. Neuroimmunol..

[22] Burman J., Raininko R., Fagius J. (2011). Bilateral and recurrent optic neuritis in multiple sclerosis. Acta Neurol. Scand..

[23] Quintana F.J., Patel B., Yeste A., Nyirenda M., Kenison J., Rahbari R., Fetco D., Hussain M., O’Mahony J., Magalhaes S. (2014). Canadian Pediatric Demyelinating Disease Network. Epitope spreading as an early pathogenic event in pediatric multiple sclerosis. Neurology.

[24] Frohman E.M., Racke M.K., Raine C.S. (2006). Multiple sclerosis—The plaque and its pathogenesis. N. Engl. J. Med..

[25] Qin Y., Duquette P., Zhang Y., Talbot P., Poole R., Antel J. (1998). Clonal expansion and somatic hypermutation of V (H) genes of B cells from cerebrospinal fluid in multiple sclerosis. J. Clin. Investig..

[26] Noseworthy J.H., Lucchinetti C., Rodriguez M., Weinshenker B.G. (2000). Multiple sclerosis. N. Engl. J. Med..

[27] Sriram S., Steiner I. (2005). Experimental allergic encephalomyelitis: A misleading model of multiple sclerosis. Ann. Neurol..

[28] Hellmann M.A., Steiner I., Mosberg-Galili R. (2011). Sudden sensorineural hearing loss in multiple sclerosis: Clinical course and possible pathogenesis. Acta Neurol. Scand..

[29] Lemus H.N., Warrington A.E., Rodriguez M. (2018). Multiple Sclerosis: Mechanisms of Disease and Strategies for Myelin and Axonal Repair. Neurol. Clin..

[30] Nancy J., Newman M.D. Atlanta, Georgia, the Optic Neuritis Treatment Trial. Commentary, AAO. https://www.aaojournal.org/article/S0161-6420(19)32364-4/pdf.

[31] Beck R.W., Gal R.L. (2008). Treatment of acute optic neuritis: A summary of findings from the optic neuritis treatment trial. Arch. Ophthalmol..

[32] Beck R.W., Cleary P.A., Anderson M.M., Keltner J.L., Shults W.T., Kaufman D.I., Buckley E.G., Corbett J.J., Kupersmith M.J., Miller N.R. (1992). A randomized controlled trail of corticosteroids in the treatment of acute optic neuritis. N. Engl. J. Med..

[33] Optic Neuritis Study Group (1997). Visual function 5 years after optic neuritis: Experience of the Optic Neuritis Treatment Trial. Arch. Ophthalmol..

[34] Gal R.L., Vedula S.S., Beck R. (2015). Corticosteroids for treating optic neuritis. Cochrane Database Syst. Rev..

[35] Petzold A., Braithwaite T., van Oosten B.W., Balk L., Martinez-Lapiscina E.H., Wheeler R., Wiegerinck N., Waters C., Plant G.T. (2020). Case for a new corticosteroid treatment trial in optic neuritis: Review of updated evidence. J. Neurol. Neurosurg. Psychiatry.

[36] Stiebel-Kalish H., Hellmann M.A., Mimouni M., Paul F., Bialer O., Bach M., Lotan I. (2019). Does time equal vision in the acute treatment of a cohort of AQP4 and MOG optic neuritis?. Neurol. Neuroimmunol. Neuroinflamm..

[37] Bsteh G., Berek K., Hegen H., Teuchner B., Buchmann A., Voortman M.M., Auer M., Zinganell A., Di Pauli F., Deisenhammer F. (2019). Serum neurofilament levels correlate with retinal nerve fiber layer thinning in multiple sclerosis. Mult. Scler. J..

[38] Osinga E., van Oosten B., de Vries-Knoppert W., Petzold A. (2017). Time is vision in recurrent optic neuritis. Brain Res..

[39] Phuljhele S., Kedar S., Saxena R. (2021). Approach to optic neuritis: An update. Indian J. Ophthalmol..

[40] Horton L., Bennett J.L. (2018). Acute Management of Optic Neuritis: An Evolving Paradigm. J. Neuroophthalmol..

[41] Wilhelm H., Schabet M. (2015). Continuing medical education the diagnosis and treatment of optic neuritis. Dtsch. Arztebl. Int..

[42] Le Page E., Veillard D., Laplaud D.A., Hamonic S., Wardi R., Lebrun C., Zagnoli F., Wiertlewski S., Deburghgraeve V., Coustans M. (2015). Oral versus intravenous high-dose methylprednisolone for treatment of relapses inpatients with multiple sclerosis (COPOUSEP): A randomised, controlled, double-blind, non-inferiority trial. Lancet.

[43] Bonnan M., Cabre P. (2012). Plasma Exchange in Severe Attacks of Neuromyelitis Optica. Mult. Scler. Int..

[44] Bennett J.L. (2019). Optic Neuritis. Continuum.

[45] Jakimovski D., Kolb C., Ramanathan M., Zivadinov R., Weinstock-Guttman B. (2018). Interferon β for Multiple Sclerosis. Cold Spring Harb. Perspect. Med..

[46] Dhib-Jalbut S., Marks S. (2010). Interferon-beta mechanisms of action in multiple sclerosis. Neurology.

[47] Rudick R.A., Ransohoff R.M., Lee J.C., Peppler R., Yu M., Mathisen P.M., Tuohy V.K. (1998). In vivo effects of interferon beta-1a on immunosuppressive cytokines in multiple sclerosis. Neurology.

[48] Hartrich L., Weinstock-Guttman B., Hall D., Badgett D., Baier M., Patrick K., Feichter J., Hong J., Ramanathan M. (2003). Dynamics of immune cell trafficking in interferon-β treated multiple sclerosis patients. J. Neuroimmunol..

[49] Rizzo F., Giacomini E., Mechelli R., Buscarinu M.C., Salvetti M., Severa M., Coccia E.M. (2016). Interferon-β therapy specifically reduces pathogenic memory B cells in multiple sclerosis patients by inducing a FAS-mediated apoptosis. Immunol. Cell. Biol..

[50] Molnarfi N., Prod’homme T., Schulze-Topphoff U., Spencer C.M., Weber M.S., Patarroyo J.C., Lalive P.H., Zamvil S.S. (2015). Glatiramer acetate treatment negatively regulates type I interferon signaling. Neurol. Neuroimmunol. Neuroinflamm..

[51] Weber M.S., Hohlfeld R., Zamvil S.S. (2007). Mechanism of action of glatiramer acetate in treatment of multiple sclerosis. Neurotherapeutics.

[52] Prod’homme T., Zamvil S.S. (2019). The Evolving Mechanisms of Action of Glatiramer Acetate. Cold Spring Harb. Perspect. Med..

[53] Polman C.H., Reingold S.C., Banwell B., Clanet M., Cohen J.A., Filippi M., Fujihara K., Havrdova E., Hutchinson M., Kappos L. (2011). Diagnostic criteria for multiple sclerosis: 2010 revisions to the McDonald criteria. Ann. Neurol..

[54] Kieseier B.C., Benamor M. (2014). Pregnancy outcomes following maternal and paternal exposure to teriflunomide during treatment for relapsing-remitting multiple sclerosis. Neurol. Ther..

[55] Andersen J.B., Moberg J.Y., Spelman T., Magyari M. (2018). Pregnancy Outcomes in Men and Women Treated with Teriflunomide. A Population-Based Nationwide Danish Register Study. Front. Immunol..

[56] Fazekas F., Lublin F.D., Li D., Freedman M.S., Hartung H.P., Rieckmann P., Sørensen P.S., Maas-Enriquez M., Sommerauer B., Hanna K. (2008). Intravenous immunoglobulin in relapsing-remitting multiple sclerosis: A dose-finding trial. Neurology.

[57] Sørensen P.S., Sellebjerg F., Lycke J., Färkkilä M., Créange A., Lund C.G., Schluep M., Frederiksen J.L., Stenager E., Pfleger C. (2016). Minocycline added to subcutaneous interferon β-1a in multiple sclerosis: Randomized Recycline study. Eur. J. Neurol..

[58] Metz L.M., Li D.K.B., Traboulsee A.L., Duquette P., Eliasziw M., Cerchiaro G., Greenfield J., Riddehough A., Yeung M., Kremenchutzky M. (2017). Trial of Minocycline in a Clinically Isolated Syndrome of Multiple Sclerosis. N. Engl. J. Med..

[59] Jacobs L.D., Beck R.W., Simon J.H., Kinkel R.P., Brownscheidle C.M., Murray T.J., Simonian N.A., Slasor P.J., Sandrock A.W., The CHAMPS Study Group (2000). Intramuscular interferon beta-1a therapy initiated during a first demyelinating event in multiple sclerosis. N. Engl. J. Med..

[60] Goodin D.S., Bates D. (2009). Treatment of early multiple sclerosis: The value of treatment initiation after a first clinical episode. Mult. Scler..

[61] Comi G., Filippi M., Barkhof F., Durelli L., Edan G., Fernández O., Hartung H., Seeldrayers P., Sørensen P.S., Rovaris M. (2001). Effect of early interferon treatment on conversion to definite multiple sclerosis: A randomised study. Lancet.

[62] Efendi H. (2015). Clinically Isolated Syndromes: Clinical Characteristics, Differential Diagnosis, and Management. Noro Psikiyatr. Ars..

[63] Marcus J.F., Waubant E.L. (2013). Updates on Clinically Isolated Syndrome and Diagnostic Criteria for Multiple Sclerosis. Neurohospitalist.

[64] Gold R., Wolinsky J.S. (2011). Pathophysiology of multiple sclerosis and the place of teriflunomide. Acta Neurol. Scand..

[65] Comi G., De Stefano N., Freedman M.S., Barkhof F., Polman C.H., Uitdehaag B.M.J., Casset-Semanaz F., Hennessy B., Moraga M.S., Rocak S. (2012). Comparison of two dosing frequencies of subcutaneous interferon beta-1a in patients with a first clinical demyelinating event suggestive of multiple sclerosis (REFLEX): A phase 3 randomised controlled trial. Lancet Neurol..

[66] Comi G., De Stefano N., Freedman M.S., Barkhof F., Uitdehaag B.M., de Vos M., Marhardt K., Chen L., Issard D., Kappos L. (2017). Subcutaneous interferon β-1a in the treatment of clinically isolated syndromes: 3-year and 5-year results of the phase III dosing frequency-blind multicentre REFLEXION study. J. Neurol. Neurosurg. Psychiatry.

[67] Miller A.E., Wolinsky J.S., Kappos L., Comi G., Freedman M.S., Olsson T.P., Bauer D., Benamor M., Truffinet P., O’Connor P.W. (2014). Oral teriflunomide for patients with a first clinical episode suggestive of multiple sclerosis (TOPIC): A randomised, double-blind, placebo-controlled, phase 3 trial. Lancet Neurol..

[68] Albert C., Mikolajczak J., Liekfeld A., Piper S.K., Scheel M., Zimmermann H.G., Nowak C., Dörr J., Bellmann-Strobl J., Chien C. (2020). Fingolimod after a first unilateral episode of acute optic neuritis (MOVING)—Preliminary results from a randomized, rater-blind, active-controlled, phase 2 trial. BMC Neurol..

[69] Cadavid D., Balcer L., Galetta S., Aktas O., Ziemssen T., Vanopdenbosch L., Frederiksen J., Skeen M., Jaffe G.J., Butzkueven H. (2017). Safety and efficacy of opicinumab in acute optic neuritis (RENEW): A randomised, placebo-controlled, phase 2 trial. Lancet Neurol..

[70] Ranger A., Ray S., Szak S., Dearth A., Allaire N., Murray R., Gardner R., Cadavid D., Mi S. (2017). Anti-LINGO-1 has no detectable immunomodulatory effects in preclinical and phase 1 studies. Neurol. Neuroimmunol. Neuroinflamm..

[71] Swanson W.B., Gong T., Zhang Z., Eberle M., Niemann D., Dong R., Rambhia K.J., Ma P.X. (2020). Controlled release of odontogenic exosomes from a biodegradable vehicle mediates dentinogenesis as a novel biomimetic pulp capping therapy. J. Control. Release.

[72] Mathew B., Torres L.A., Gamboa Acha L., Tran S., Liu A., Patel R., Chennakesavalu M., Aneesh A., Huang C.C., Feinstein D.L. (2021). Uptake and distribution of administered bone marrow mesenchymal stem cell extracellular vesicles in retina. Cells.

[73] Mathew B., Ravindran S., Liu X., Torres L., Chennakesavalu M., Huang C.C., Feng L., Zelka R., Lopez J., Sharma M. (2019). Mesenchymal stem cell-derived extracellular vesicles and retinal ischemia-reperfusion. Biomaterials.

[74] Dai Y.D., Sheng H., Dias P., Jubayer Rahman M., Bashratyan R., Regn D., Marquardt K. (2017). Autoimmune responses to exosomes and candidate antigens contribute to type 1 diabetes in non-obese diabetic mice. Curr. Diabetes Rep..

[75] Laso-Garcia F., Ramos-Cejudo J., Carrillo-Salinas F.J., Otero-Ortega L., Feliu A., Gomez-de Frutos M., Mecha M., Díez-Tejedor E., Guaza C., Gutiérrez-Fernández M. (2018). Therapeutic potential of extracellular vesicles derived from human mesenchymal stem cells in a model of progressive multiple sclerosis. PLoS ONE.

[76] Thomi G., Joerger-Messerli M., Haesler V., Muri L., Surbek D., Schoeberlein A. (2019). Intranasally administered exosomes from umbilical cord stem cells have preventive neuroprotective effects and contribute to functional recovery after perinatal brain injury. Cells.

[77] Duffy C.P., McCoy C.E. (2020). The role of MicroRNAs in repair processes in multiple sclerosis. Cells.

[78] Ma Q., Matsunaga A., Ho B., Oksenberg J.R., Didonna A. (2020). Oligodendrocyte specific Argonaute profiling identifies microRNAs associated with experimental autoimmune encephalomyelitis. J. Neuroinflamm..

[79] Marangon D., Boda E., Parolisi R., Negri C., Giorgi C., Montarolo F., Perga S., Bertolotto A., Buffo A., Abbracchio M.P. (2020). In vivo silencing of miR-125a-3p promotes myelin repair in models of white matter demyelination. Glia.

[80] Lennon V.A., Wingerchuk D.M., Kryzer T.J., Pittock S.J., Lucchinetti C.F., Fujihara K., Nakashima I., Weinshenker B.G. (2004). A serum autoantibody marker of neuromyelitis optica: Distinction from multiple sclerosis. Lancet.

[81] Misu T., Fujihara K., Kakita A., Konno H., Nakamura M., Watanabe S., Takahashi T., Nakashima I., Takahashi H., Itoyama Y. (2007). Loss of aquaporin 4 in lesions of neuromyelitis optica: Distinction from multiple sclerosis. Brain.

[82] Roemer S.F., Parisi J.E., Lennon V.A., Benarroch E.E., Lassmann H., Bruck W., Mandler R.N., Weinshenker B.G., Pittock S.J., Wingerchuk D.M. (2007). Pattern-specific loss of aquaporin-4 immunoreactivity distinguishes neuromyelitis optica from multiple sclerosis. Brain.

[83] Kawachi I., Lassmann H. (2017). Neurodegeneration in multiple sclerosis and neuromyelitis optica. J. Neurol. Neurosurg. Psychiatry.

[84] Hinson S.R., Romero M.F., Popescu B.F., Lucchinetti C.F., Fryer J.P., Wolburg H., Fallier-Becker P., Noell S., Lennon V.A. (2012). Molecular outcomes of neuromyelitis optica (NMO)-IgG binding to aquaporin-4 in astrocytes. Proc. Natl. Acad. Sci. USA.

[85] Crane J.M., Lam C., Rossi A., Gupta T., Bennett J.L., Verkman A.S. (2011). Binding affinity and specificity of neuromyelitis optica autoantibodies to aquaporin-4 M1/M23 isoforms and orthogonal arrays. J. Biol. Chem..

[86] Papadopoulos M.C., Verkman A.S. (2012). Aquaporin 4 and neuromyelitis optica. Lancet Neurol..

[87] Contentti E.C., Correale J. (2021). Neuromyelitis optica spectrum disorders: From pathophysiology to therapeutic strategies. J. Neuroinflamm..

[88] Stiebel-Kalish H., Lotan I., Brody J., Chodick G., Bialer O., Marignier R., Bach M., Hellmann M.A. (2017). Retinal nerve fiber layer may be better preserved in MOG-IgG versus AQP4-IgG optic neuritis: A cohort study. PLoS ONE.

[89] Chen J.J., Tobin W.O., Majed M., Jitprapaikulsan J., Fryer J.P., Leavitt J.A., Flanagan E.P., McKeon A., Pittock S.J. (2018). Prevalence of myelin oligodendrocyte glycoprotein and aquaporin-4-IgG in patients in the optic neuritis treatment trial. JAMA Ophthalmol..

[90] Kleiter I., Gahlen A., Borisow N., Fischer K., Wernecke K.D., Wegner B., Hellwig K., Pache F., Ruprecht K., Havla J. (2016). Neuromyelitis optica: Evaluation of 871 attacks and 1,153 treatment courses. Ann. Neurol..

[91] Deschamps R., Gueguen A., Parquet N., Saheb S., Driss F., Mesnil M., Vignal C., Aboab J., Depaz R., Gout O. (2016). Plasma exchange response in 34 patients with severe optic neuritis. J. Neurol..

[92] Restrepo-Aristizábal C., Giraldo L.M., Giraldo Y.M., Pino-Pérez A.M., Álvarez-Gómez F., Franco C.A., Tobón J.V., Ascencio J.L., Zuluaga M.I. (2021). PLEX: The best first-line treatment in nmosd attacks experience at a single center in Colombia. Heliyon.

[93] Yasuda T., Mikami T., Kawase Y. (2015). Efficacy of tryptophan immunoadsorption plasmapheresis for neuromyelitis optica in two cases. Ther. Apher. Dial..

[94] Bonnan M., Valentino R., Debeugny S., Merle H., Fergé J.L., Mehdaoui H., Cabre P. (2018). Short delay to initiate plasma exchange is the strongest predictor of outcome in severe attacks of NMO spectrum disorders. J. Neurol. Neurosurg. Psychiatry.

[95] Kleiter I., Gahlen A., Borisow N., Fischer K., Wernecke K.D., Hellwig K., Pache F., Ruprecht K., Havla J., Kümpfel T. (2018). Apheresis therapies for NMOSD attacks. A retrospective study of 207 therapeutic interventions. Neurol. Neurol. Neuroimmunol. Neuroinflamm..

[96] Oji S., Nomura K. (2017). Immunoadsorption in neurological disorders. Transfus. Apher. Sci..

[97] Schwartz J. (2016). Guidelines on the use of therapeutic apheresis in clinical practice—Evidence-based approach from the writing committee of the American society for apheresis: The seventh special issue. J. Clin. Apher..

[98] Lipphardt M., Wallbach M., Koziolek M.J. (2020). Plasma exchange or immunoadsorption in demyelinating diseases: A meta-analysis. J. Clin. Med..

[99] Li X., Tian D.C., Fan M., Xiu Y., Wang X., Li T., Jia D., Xu W., Song T., Shi F.D. (2020). Intravenous immunoglobulin for acute attacks in neuromyelitis optica spectrum disorders (NMOSD). Mult. Scler. Relat. Disord..

[100] Greenberg B.M., Thomas K.P., Krishnan C., Kaplin A.I., Calabresi P.A., Kerr D.A. (2007). Idiopathic transverse myelitis: Corticosteroids, plasma exchange, or cyclophosphamide. Neurology.

[101] Carnero Contentti E., Rojas J.I., Cristiano E., Daccach Marques V., Flores-Rivera J., Lana-Peixoto M., Carlos N., Papais-Alvarenga R., Sato D.K., de Castillo I.S. (2020). Latin American consensus recommendations for management and treatment of neuromyelitis optica spectrum disorders in clinical practice. Mult. Scler. Relat. Disord..

[102] Tugizova M., Vlahovic L., Tomczak A., Wetzel N.S., Han M.H. (2021). New Therapeutic Landscape in Neuromyelitis Optica. Curr. Treat Options Neurol..

[103] Held F., Klein A.-K., Berthele A. (2021). Drug Treatment of Neuromyelitis Optica Spectrum Disorders: Out with the Old, in with the New?. ImmunoTargets Ther..

[104] Sharrack B., Saccardi R., Alexander T., Badoglio M., Burman J., Farge D., Greco R., Jessop H., Kazmi M., Kirgizov K. (2020). Autologous haematopoietic stem cell transplantation and other cellular therapy in multiple sclerosis and immune-mediated neurological diseases: Updated guidelines and recommendations from the EBMT Autoimmune Diseases Working Party (ADWP) and the Joint Accreditation Committee of EBMT and ISCT (JACIE). Bone Marrow Transplant..

[105] Ceglie G., Papetti L., Valeriani M., Merli P. (2020). Hematopoietic stem cell transplantation in neuromyelitis optica-spectrum disorders (NMO-SD): State-of-the-art and future perspectives. Int. J. Mol. Sci..

[106] Chan K.-H., Lee C.-Y. (2021). Treatment of Neuromyelitis Optica Spectrum Disorders, Review. Int. J. Mol. Sci..

[107] Wallach A.I., Tremblay M., Kister I. (2021). Advances in the Treatment of Neuromyelitis Optica Spectrum Disorder. Neurol. Clin..

[108] Miljkovic D., Samardzic T., Drakulic D., Stosic-Grujicic S., Trajkovic V. (2002). Immunosuppressants leflunomide and mycophenolic acid inhibit fibroblast IL-6 production by distinct mechanisms. Cytokine.

[109] Allison A.C., Eugui E.M. (2000). Mycophenolate mofetil and its mechanisms of action. Immunopharmacology.

[110] Nielsen O.H., Vainer B., Rask-Madsen J. (2001). Review article: The treatment of inflammatory bowel disease with 6-mercaptopurine or azathioprine. Aliment. Pharmacol. Ther..

[111] Bichuetti D.B., Perin M.M.M., Souza N.A., Oliveira E.M.L. (2019). Treating neuromyelitis optica with azathioprine: 20-year clinical practice. Mult. Scler..

[112] Jacob A., Matiello M., Weinshenker B.G., Wingerchuk D.M., Lucchinetti C., Shuster E., Carter J., Keegan B.M., Kantarci O.H., Pittock S.J. (2009). Treatment of neuromyelitis optica with mycophenolate mofetil: Retrospective analysis of 24 patients. Arch. Neurol..

[113] Montcuquet A., Collongues N., Papeix C., Zephir H., Audoin B., Laplaud D., Bourre B., Brochet B., Camdessanche J.P., Labauge P. (2017). Effectiveness of mycophenolate mofetil as first-line therapy in AQP4-IgG, MOG-IgG, and seronegative neuromyelitis optica spectrum disorders. Mult. Scler..

[114] Songwisit S., Kosiyakul P., Jitprapaikulsan J., Prayoonwiwat N., Ungprasert P., Siritho S. (2020). Efficacy and safety of mycophenolate mofetil therapy in neuromyelitis optica spectrum disorders: A systematic review and meta-analysis. Sci. Rep..

[115] Collongues N., Ayme-Dietrich E., Monassier L., de Seze J. (2019). Pharmacotherapy for Neuromyelitis Optica Spectrum Disorders: Current Management and Future Options. Drugs.

[116] Kowarik M.C., Soltys J., Bennett J.L. (2014). The treatment of neuromyelitis optica. J. Neuroophthalmol..

[117] Enriquez C.A.G., Espiritu A.I., Pasco P.M.D. (2019). Efficacy and tolerability of mitoxantrone for neuromyelitis optica spectrum disorder: A systematic review. J. Neuroimmunol..

[118] Jarius S., Aboul-Enein F., Waters P., Kuenz B., Hauser A., Berger T., Lang W., Reindl M., Vincent A., Kristoferitsch W. (2008). Antibody to aquaporin-4 in the long-term course of neuromyelitis optica. Brain.

[119] Bichuetti D.B., Oliveira E.M., Boulos Fde C., Gabbai A.A. (2012). Lack of response to pulse cyclophosphamide in neuromyelitis optica: Evaluation of 7 patients. Arch. Neurol..

[120] Xu Y., Wang Q., Ren H.T., Qiao L., Zhang Y., Fei Y.Y. (2016). Comparison of efficacy and tolerability of azathioprine, mycophenolate mofetil, and cyclophosphamide among patients with neuromyelitis optica spectrum disorder: A prospective cohort study. J. Neurol. Sci..

[121] Chen B., Wu Q., Ke G., Bu B. (2017). Efficacy and safety of tacrolimus treatment for neuromyelitis optica spectrum disorder. Sci. Rep..

[122] Trebst C., Jarius S., Berthele A., Paul F., Schippling S., Wildemann B., Borisow N., Kleiter I., Aktas O., Kümpfel T. (2014). Update on the diagnosis and treatment of neuromyelitis optica: Recommendations of the Neuromyelitis Optica Study Group (NEMOS). J. Neurol..

[123] Kim S.H., Jeong I.H., Hyun J.W., Joung A., Jo H.J., Hwang S.H., Yun S., Joo J., Kim H.J. (2015). Treatment outcomes with rituximab in 100 patients with neuromyelitis optica: Influence of FCGR3A polymorphisms on the therapeutic response to rituximab. JAMA Neurol..

[124] Ellwardt E., Ellwardt L., Bittner S., Zipp F. (2018). Monitoring Bcell repopulation after depletion therapy in neurologic patients. Neurol. Neuroimmunol. Neuroinflamm..

[125] Tahara M., Oeda T., Okada K., Kiriyama T., Ochi K., Maruyama H., Fukaura H., Nomura K., Shimizu Y., Mori M. (2020). Safety and efficacy of rituximab in neuromyelitis optica spectrum disorders (RIN-1 study): A multicentre, randomised, double-blind, placebo-controlled trial. Lancet Neurol..

[126] Graf J., Mares J., Barnett M., Aktas O., Albrecht P., Zamvil S.S., Hartung H.P. (2020). Targeting B cells to modify MS, NMOSD, and MOGAD: Part 2. Neurol. Neuroimmunol. Neuroinflamm..

[127] Marcinnò A., Marnetto F., Valentino P., Martire S., Balbo A., Drago A., Leto M., Capobianco M., Panzica G., Bertolotto A. (2018). Rituximab-induced hypogammaglobulinemia in patients with neuromyelitis optica spectrum disorders. Neurol. Neuroimmunol. Neuroinflamm..

[128] Wingerchuk, AAN2019. https://issuu.com/americanacademyofneurology/docs/aan_onsiteguide_web_with_links.

[129] Ghrenassia E., Mariotte E., Azoulay E. (2018). Rituximab-related severe toxicity. Int. J. Crit. Care Emerg. Med..

[130] Holmøy T., Høglund R.A., Illes Z., Myhr K.-M., Torkildsen Ø. (2021). Recent progress in maintenance treatment of neuromyelitis optica spectrum disorder. J. Neurol..

[131] Chihara N., Aranami T., Sato W., Miyazaki Y., Miyake S., Okamoto T., Ogawa M., Toda T., Yamamura T. (2011). Interleukin 6 signaling promotes anti-aquaporin 4 autoantibody production from plasma blasts in neuromyelitis optica. Proc. Natl. Acad. Sci. USA.

[132] Zhang C., Zhang M., Qiu W., Ma H., Zhang X., Zhu Z., Yang C.S., Jia D., Zhang T.X., Yuan M. (2020). Safety and efficacy of tocilizumab versus azathioprine in highly relapsing neuromyelitis optica spectrum disorder (TANGO): An open-label, multicentre, randomised, phase 2 trial. Lancet Neurol..

[133] Agasing A.M., Wu Q., Khatri B., Borisow N., Ruprecht K., Brandt A.U., Gawde S., Kumar G., Quinn J.L., Ko R.M. (2020). Transcriptomics and proteomics reveal a cooperation between interferon and T-helper 17 cells in neuromyelitis optica. Nat. Commun..

[134] Pardo S., Giovannoni G., Hawkes C., Lechner-Scott J., Waubant E., Levy M. (2019). Editorial on: Eculizumab in aquaporin-4-positive neuromyelitis optica spectrum disorder. Mult. Scler. Relat. Disord..

[135] Levy M., Fujihara K., Palace J. (2021). New therapies for neuromyelitis optica spectrum disorder. Lancet Neurol..

[136] Fox E., Lovett-Racke A.E., Gormley M., Liu Y., Petracca M., Cocozza S., Shubin R., Wray S., Weiss M.S., Bosco J.A. (2021). A phase 2 multicenter study of ublituximab, a novel glycoengineered anti-CD20 monoclonal antibody, in patients with relapsing forms of multiple sclerosis. Mult. Scler..

[137] Pittock S.J., Berthele A., Fujihara K., Nakashima I., Kim H.J., Levy M., Palace J., Nakashima I., Terzi M., Totolyan N. (2019). Eculizumab in aquaporin-4-positive neuromyelitis optica spectrum disorder. N. Engl. J. Med..

[138] Tullman M.J., Zabeti A., Vuocolo S., Dinh Q. (2021). Inebilizumab for treatment of neuromyelitis optica spectrum disorder. Neurodegener. Dis. Manag..

[139] Cree B.A.C., Bennett J.L., Kim H.J., Weinshenker B.G., Pittock S.J., Wingerchuk D.M., Fujihara K., Paul F. (2019). Inebilizumab for the treatment of neuromyelitis optica spectrum disorder (N-MOmentum): A double-blind, randomized placebo-controlled phase 2/3 trial. Lancet.

[140] Cree B.A., Bennett J.L., Kim H.J., Weinshenker B.G., Pittock S.J., Wingerchuk D., Fujihara K., Paul F., Cutter G.R., Marignier R. (2021). Sensitivity analysis of the primary endpoint from the N-MOmentum study of inebilizumab in NMOSD. Mult. Scler..

[141] Marignier R., Bennett J.L., Kim H.J., Weinshenker B.G., Pittock S.J., Wingerchuk D., Fujihara K., Paul F., Cutter G.R., Green A.J. (2021). Disability outcomes in the N-MOmentum trial of inebilizumab in neuromyelitis optica spectrum disorder. Neurol. Neuroimmunol. Neuroinflamm..

[142] Traboulsee A., Greenberg B.M., Bennett J.L., Szczechowski L., Fox E., Shkrobot S., Yamamura T., Terada Y., Kawata Y., Wright P. (2020). Safety and efficacy of satralizumab monotherapy in neuromyelitis optica spectrum disorder: A randomised, double-blind, multicentre, placebo-controlled phase 3 trial. Lancet Neurol..

[143] Yamamura T., Kleiter I., Fujihara K., Palace J., Greenberg B., Zakrzewska-Pniewska B., Patti F., Tsai C.-P., Saiz A., Yamazaki H. (2019). Trial of Satralizumab in neuromyelitis optica spectrum disorder. N. Engl. J. Med..

[144] (2020). Enspryng Prescribing Information. https://www.gene.com/download/pdf/enspryng_prescribing.pdf.

[145] Mealy M.A., Levy M. (2019). A pilot safety study of ublituximab, a monoclonal antibody against CD20, in acute relapses of neuromyelitis optica spectrum disorder. Medicine.

[146] Kim W., Kim H.J. (2020). Monoclonal antibody therapies for multiple sclerosis and neuromyelitis optica spectrum disorder. J. Clin. Neurol..

[147] Tradtrantip L., Asavapanumas N., Verkman A.S. (2020). Emerging therapeutic targets for neuromyelitis optica spectrum disorder. Expert Opin. Ther. Targets.

[148] Liossis S.N., Staveri C. (2021). What’s new in the treatment of systemic lupus erythematosus. Front. Med..

[149] Zhang C., Tian D.-C., Yang C.-S., Han B., Wang J., Yang L., Shi F.-D. (2017). Safety and efficacy of bortezomib in patients with highly relapsing neuromyelitis optica spectrum disorder. JAMA Neurol..

[150] Howard J.F., Bril V., Burns T.M., Mantegazza R., Bilinska M., Szczudlik A., Beydoun S., Garrido F.J.R.R., Piehl F., Rottoli M. (2019). Randomized phase 2 study of FcRn antagonist efgartigimod in generalized myasthenia gravis. Neurology.

[151] Montalban X., Arnold D.L., Weber M.S., Staikov I., Piasecka-Stryczynska K., Willmer J., Martin E.C., Dangond F., Syed S., Wolinsky J.S. (2019). Placebo-Controlled Trial of an Oral BTK Inhibitor in Multiple Sclerosis. N. Engl. J. Med..

[152] Shimizu F., Schaller K.L., Owens G.P., Cotleur A.C., Kellner D., Takeshita Y., Obermeier B., Kryzer T.J., Sano Y., Kanda T. (2017). Glucose-regulated protein 78 autoantibody associates with blood-brain barrier disruption in neuromyelitis optica. Sci. Transl. Med..

[153] Lee J.W., de Fontbrune F.S., Lee L.W.L., Pessoa V., Gualandro S., Füreder W., Ptushkin V., Rottinghaus S.T., Volles L., Shafner L. (2019). Ravulizumab (ALXN1210) vs eculizumab in adult patients with PNH naive to complement inhibitors: The 301 study. Blood.

[154] Kulasekararaj A.G., Hill A., Rottinghaus S.T., Langemeijer S., Wells R., Gonzalez-Fernandez F.A., Gaya A., Lee J.W., Gutierrez E.O., Piatek C.I. (2019). Ravulizumab (ALXN1210) vs eculizumab in C5-inhibitor–experienced adult patients with PNH: The 302 study. Blood.

[155] McKeage K. (2019). Ravulizumab: First global approval. Drugs.

[156] Araki M., Yamamura T. (2017). Neuromyelitis optica spectrum disorders: Emerging therapies. Clin. Exp. Neuroimmunol..

[157] Katz Sand I., Fabian M.T., Telford R., Kraus T.A., Chehade M., Masilamani M., Moran T., Farrell C., Ebel S., Cook L.J. (2018). Open-label, add-on trial of cetirizine for neuromyelitis optica. Neurol. Neuroimmunol Neuroinflamm..

[158] Roufosse F. (2018). Targeting the interleukin-5 pathway for treatment of eosinophilic conditions other than asthma. Front. Med..

[159] Tradtrantip L., Zhang H., Saadoun S., Phuan P.W., Lam C., Papadopoulos M.C., Bennett J.L., Verkman A.S. (2012). Anti-aquaporin-4 monoclonal antibody blocker therapy for neuromyelitis optica. Ann. Neurol..

[160] Derdelinckx J., Reynders T., Wens I., Cools N., Willekens B. (2021). Cells to the Rescue: Emerging Cell-Based Treatment Approaches for NMOSD and MOGAD. Int. J. Mol. Sci..

[161] Peng F., Qiu W., Li J., Hu X., Huang R., Lin D., Bao J., Jiang Y., Bian L. (2010). A preliminary result of treatment of neuromyelitis optica with autologous peripheral hematopoietic stem cell transplantation. Neurologist.

[162] Greco R., Bondanza A., Vago L., Moiola L., Rossi P., Furlan R., Martino G., Radaelli M., Martinelli V., Carbone M.R. (2014). Allogeneic hematopoietic stem cell transplantation for neuromyelitis optica. Ann. Neurol..

[163] Burt R.K., Balabanov R., Han X., Burns C., Gastala J., Jovanovic B., Helenowski I., Jitprapaikulsan J., Fryer J.P., Pittock S.J. (2019). Autologous nonmyeloablative hematopoietic stem cell transplantation for neuromyelitis optica. Neurology.

[164] Zhang P., Liu B. (2020). Effect of autologous hematopoietic stem cell transplantation on multiple sclerosis and neuromyelitis optica spectrum disorder: A PRISMA compliant meta-analysis. Bone Marrow Transplant..

[165] Hau L., Kállay K., Kertész G., Goda V., Kassa C., Horváth O., Liptai Z., Constantin T., Kriván G. (2020). Allogeneic Haematopoietic Stem Cell Transplantation in a Refractory Case of Neuromyelitis Optica Spectrum Disorder. Mult. Scler. Relat. Disord..

[166] Zubizarreta I., Flórez-Grau G., Vila G., Cabezón R., España C., Andorra M., Saiz A., Llufriu S., Sepulveda M., Sola-Valls N. (2019). Immune Tolerance in Multiple Sclerosis and Neuromyelitis Optica with Peptide-Loaded Tolerogenic Dendritic Cells in a Phase 1b Trial. Proc. Natl. Acad. Sci. USA.

[167] Lu Z., Zhu L., Liu Z., Wu J., Xu Y., Zhang C.J. (2020). IV/IT hUC-MSCs Infusion in RRMS and NMO: A 10-Year Follow-Up Study. Front. Neurol..

[168] Yao X., Su T., Verkman A.S. (2016). Clobetasol promotes remyelination in a mouse model of neuromyelitis optica. Acta Neuropathol. Commun..

[169] Sepúlveda M., Armangué T., Sola-Valls N., Arrambide G., Meca-Lallana J.E., Oreja-Guevara C., Mendibe M., De Arcaya A.A., Aladro Y., Casanova B. (2016). Neuromyelitis optica spectrum disorders comparison according to the phenotype and serostatus. Neurol. Neuroimmunol. Neuroinflamm..

[170] Mealy M.A., Kim S.H., Schmidt F., López R., Jimenez Arango J.A., Paul F., Wingerchuk D.M., Greenberg B.M., Kim H.J., Levy M. (2018). Aquaporin-4 serostatus does not predict response to immunotherapy in neuromyelitis optica spectrum disorders. Mult. Scler. J..

[171] Quarles R.H. (2002). Myelin Sheaths: Glycoproteins Involved in Their Formation, Maintenance and Degeneration. Cell. Mol. Life Sci..

[172] Ambrosius W., Michalak S., Kozubski W., Kalinowska A. (2020). Myelin Oligodendrocyte Glycoprotein Antibody-Associated Disease: Current Insights into the Disease Pathophysiology, Diagnosis and Management. Int. J. Mol. Sci..

[173] Sharpe A.H., Freeman G.J. (2002). The B7-CD28 Superfamily. Nat. Rev. Immunol..

[174] Höftberger R., Guo Y., Flanagan E.P., Lopez-Chiriboga A.S., Endmayr V., Hochmeister S., Joldic D., Pittock S.J., Tillema J.M., Gorman M. (2020). The pathology of central nervous system inflammatory demyelinating disease accompanying myelin oligodendrocyte glycoprotein autoantibody. Acta Neuropathol..

[175] Ramanathan S., Mohammad S., Tantsis E., Nguyen T.K., Merheb V., Fung V.S.C., White O.B., Broadley S., Lechner-Scott J., Vucic S. (2018). Clinical course, therapeutic responses and outcomes in relapsing MOG antibody-associated demyelination. J. Neurol. Neurosurg. Psychiatry.

[176] Whittam D.H., Karthikeayan V., Gibbons E., Kneen R., Chandratre S., Ciccarelli O., Hacohen Y., de Seze J., Deiva K., Hintzen R.Q. (2020). Treatment of MOG antibody associated disorders: Results of an international survey. J. Neurol..

[177] Chen J.J., Flanagan E.P., Bhatti M.T., Jitprapaikulsan J., Dubey D., Lopez Chiriboga A.S.S., Fryer J.P., Weinshenker B.G., McKeon A., Tillema J.M. (2020). Steroid-sparing maintenance immunotherapy for MOG-IgG associated disorder. Neurology.

[178] Song H., Zhou H., Yang M., Wang J., Liu H., Sun M., Xu Q., Wei S. (2019). Different characteristics of aquaporin-4 and myelin oligodendrocyte glycoprotein antibody-seropositive male optic neuritis in China. J. Ophthalmol..

[179] Hacohen Y., Wong Y.Y., Lechner C., Jurynczyk M., Wright S., Konuskan B., Kalser J., Poulat A.L., Maurey H., Ganelin-Cohen E. (2018). Disease course and treatment responses in children with relapsing myelin oligodendrocyte glycoprotein antibody-associated disease. JAMA Neurol..

[180] Lu Q., Luo J., Hao H., Liu R., Jin H., Jin Y., Gao F. (2020). Efficacy and safety of long-term immunotherapy in adult patients with MOG antibody disease: A systematic analysis. J. Neurol..

[181] Li S., Ren H., Yan X., Xu T., Zhang Y., Yin H., Zhang W., Li J., Ren X., Fang F. (2020). Long-term efficacy of mycophenolate mofetil in myelin oligodendrocyte glycoprotein antibody-associated disorders: A prospective study. Neurol. Neuroimmunol. Neuroinflamm..

[182] Whittam D.H., Cobo-Calvo A., Lopez-Chiriboga A.S., Pardo S., Dodd J., Brandt A., Berek K., Berger T., Gombolay G., Oliveira L.M. (2018). Treatment of MOG-IgG associated demyelination with Rituximab: A multinational study of 98 patients. Neurology.

[183] Whittam D.H., Cobo-Calvo A., Lopez-Chiriboga A.S., Pardo S., Gornall M., Cicconi S., Brandt A., Berek K., Berger T., Jelcic I. (2020). Treatment of MOG-IgG-associated disorder with rituximab: An international study of 121 patients. Mult. Scler. Relat. Disord..

[184] Contentti E.C., Marrodan M., Correale J. (2021). Emerging drugs for the treatment of adult MOGIgG-associated diseases. Expert Opin. Emerg. Drugs.

[185] Renjen P.N., Chaudhari D.M., Ahmad K., Garg S., Mishra A. (2020). A review of chronic relapsing inflammatory optic neuropathy. Apollo Med..

[186] Kidd D., Burton B., Plant G.T., Graham E.M. (2003). Chronic relapsing inflammatory optic neuropathy (CRION). Brain.

[187] Lee H.-J., Kim B., Waters P., Woodhall M., Irani S., Ahn S., Kim S.-J., Kim S.-M. (2018). Chronic relapsing inflammatory optic neuropathy (CRION): A manifestation of myelin oligodendrocyte glycoprotein antibodies. J. Neuroinflamm..

[188] Mukharesh L., Douglas V.P., Chwalisz B.K. (2021). Chronic Relapsing Inflammatory Optic Neuropathy (CRION). Curr. Opin. Ophthalmol..

